# Progressing, not regressing: A possible solution to the problem of regression to the mean in unconscious processing studies

**DOI:** 10.3758/s13423-023-02326-x

**Published:** 2023-08-01

**Authors:** Itay Yaron, Yoav Zeevi, Uri Korisky, William Marshall, Liad Mudrik

**Affiliations:** 1 The Sagol School of Neuroscience, Tel Aviv University, Tel Aviv, 39040, Israel; 2 Department of Statistics and Operations Research, Tel Aviv University, Tel Aviv, 39040, Israel; 3 School of Psychological Sciences, Tel Aviv University, Tel Aviv, 39040, Israel; 4 Department of Psychiatry, University of Wisconsin-Madison, Madison, WI 53719 USA; 5 Department of Mathematics and Statistics, Brock University, St. Catharines, ON L2S 3A1 Canada

**Keywords:** Unconscious processing, Regression to the mean, Power, Consciousness, Reliability

## Abstract

How convincing is current evidence for unconscious processing? Recently, a major criticism suggested that some, if not much, of this evidence might be explained by a mere statistical phenomenon: regression to the mean (RttM). Excluding participants based on an awareness assessment is a common practice in studies of unconscious processing, and this post hoc data selection might lead to false effects that are driven by RttM for aware participants wrongfully classified as unaware. Here, we examined this criticism using both simulations and data from 12 studies probing unconscious processing (35 effects overall). In line with the original criticism, we confirmed that the reliability of awareness measures in the field is concerningly low. Yet, using simulations, we showed that reliability measures might be unsuitable for estimating error in awareness measures. Furthermore, we examined other solutions for assessing whether an effect is genuine or reflects RttM; all suffered from substantial limitations, such as a lack of specificity to unconscious processing, lack of power, or unjustified assumptions. Accordingly, we suggest a new nonparametric solution, which enjoys high specificity and relatively high power. Together, this work emphasizes the need to account for measurement error in awareness measures and evaluate its consequences for unconscious processing effects. It further suggests a way to meet the important challenge posed by RttM, in an attempt to establish a reliable and robust corpus of knowledge in studying unconscious processing.

**Supplementary information:**

The online version contains supplementary material available at 10.3758/s13423-023-02326-x.

Few topics in the history of cognitive science have evoked so much controversy and debate as the study of unconscious processing (for recent discussions, see Moors & Hesselmann, 2017; Newell & Shanks, 2014; Peters et al., 2017; Rothkirch & Hesselmann, 2017; Rothkirch et al., 2022; Shanks, 2017). The main disagreement concerns the extent to which participants’ behavior is affected by information that is not accessible to them (e.g., information rendered invisible using some psychophysical method; for reviews, see Breitmeyer, 2015; Kim & Blake, 2005). In fact, when reviewing the history of the field, one might conclude that it is going in circles; at each iteration, strong claims are made about the scope of unconscious processing, which are then followed by methodological criticisms of some sort, questioning the validity of these claims (for a description of this process with respect to unconscious semantic processing, see Kouider & Dehaene, 2007). More recently, a surge of findings reporting remarkably complicated unconscious processes (Mudrik et al., 2011; Sklar et al., 2012; Van Opstal, Calderon, et al., 2011a) was followed by a wave of replication failures (Biderman & Mudrik, 2017; Moors et al., 2016; Moors & Hesselmann, 2017; Stein et al., 2020; Zerweck et al., 2021) and methodological criticism (e.g., Meyen et al., 2022; Rothkirch & Hesselmann, 2017; Rothkirch et al., 2022; Schmidt, 2015; Shanks, 2017). And so, since its inception (see again Kouider & Dehaene, 2007), the field has been characterized by pendulum-like oscillations between assigning high-level functions to unconscious processes and suggesting that they strongly depend on conscious processing.

A prominent line of criticism that has captured substantial attention (Leganes-Fonteneau et al., 2021; Rothkirch et al., 2022; Shanks, 2017; Vadillo et al., 2022), and justly so, concerns Regression to the Mean (RttM), and how it might account for reported effects of unconscious processing (Shanks, 2017). This criticism targets the common practice of excluding participants who were aware of the presented stimuli (or trials where participants report such awareness), in an attempt to distil unconscious processing and avoid a situation where a group-level effect stems from participants who consciously processed the stimuli. Participant exclusion is typically done by setting some threshold for awareness; for example, if the participants are asked to perform an explicit judgment on the suppressed stimulus (e.g., determine whether an arrow points to the right or the left; an *objective measure* of awareness), the researchers might set a threshold of accuracy (e.g., a threshold of 0.65 when the chance probability is 0.5) and exclude all participants who score higher than the threshold. Such use of an objective measure of awareness to exclude participants is fairly common in the field (e.g., N. Biderman & Mudrik, 2017; Hesselmann et al., 2016; Huang et al., 2014, p. 10214; Kiefer, 2019; Rowe et al., 2020; Sklar et al., 2012; Stein et al., 2020).^1^

Yet as clearly demonstrated by Shanks (2017), such post hoc data selection is biased by RttM. An unaware subgroup is selected based on the *observed* scores in the objective awareness measure. By definition, these observed results are the combination of the *true* awareness scores and some *measurement error*. Critically, this measurement error is correlated with the observed score. That is, larger observed scores tend to have positive errors—overestimating awareness—while smaller observed scores will have negative errors—underestimating awareness (see again, Shanks, 2017, and Shanks et al., 2021, for a full explanation of this point). And so, it is likely that some of the participants who are classified as unaware are in fact aware: since we are only selecting the lower scores (i.e., participants who are below threshold), it is expected that these scores are more driven by negative errors that “push them” below the threshold, even though their true score is actually above the threshold. While the *awareness scores* of these misclassified participants make them seem unaware, their *effect scores*—that is, their performance on the task aimed at assessing whether unconscious processing took place—will be high, reflecting the fact that they were truly aware of the stimulus (for an illustration of this point, see Fig. 1). Thus, any evidence for unconscious processing from such a post hoc selected group might be due to aware participants who were misclassified as unaware. Indeed, using simulations Shanks (2017) has shown that in the absence of any unconscious effect, such RttM can lead to the false finding of unconscious processing.

**Fig. 1:**
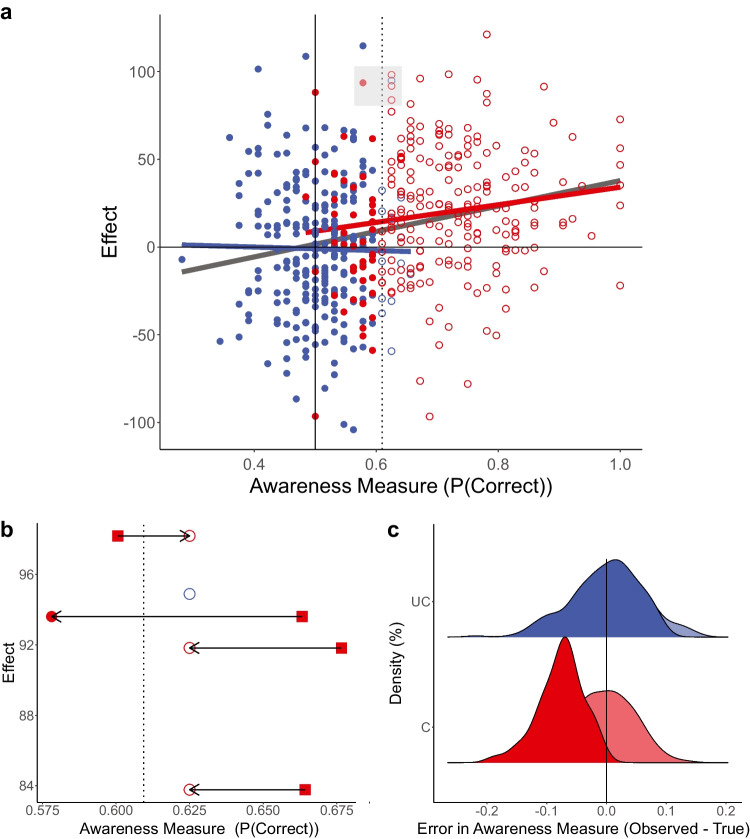
Simulated data illustrating linear relations between the effect (*y*-axis) and awareness performance (*x*-axis) in a combined sample where half the participants are truly unaware (blue) and half are truly aware (red). **a** Each circle represents the observed scores of an individual participant, which is the sum of their true scores and the measurement error. Filled circles indicate included participants. Empty circles depict the observed scores of excluded participants. Regression lines are depicted for the entire sample (gray), for the unaware participants (blue), and for the aware ones (red). **b** A zoomed-in view on the gray rectangle in Panel **a**. Black arrows links the observed (circles) and true (squares) awareness scores of each participant, respectively. **c** The distribution of the measurement errors only is presented in blue (unaware participants) and red (aware participants), both for the entire sample (nonopaque) and for the included sample only (opaque). This further demonstrates that while the measurement error for the included unaware participants is practically equally distributed around zero, this is not the case for the aware participants who were included (as their observed scores were lower than the inclusion threshold, due to measurement error). For these participants, errors are highly biased to negative values, leading to RttM. (Color figure online)

Notably, the magnitude of RttM depends on the strength of the relationship between variables (e.g., their correlation). This highlights the issue of measure reliability; the larger the errors are (i.e., the less reliable the measure is),^2^ the more aware participants will be pushed below the threshold. Thus, using error-free awareness measures is one way to reduce the impact of RttM.^3^ However, measurement errors were shown to be large for cognitive measures in general (Huber et al., 2019), and with respect to implicit processing in particular (Vadillo et al., 2022). Below, we also show that this is especially prominent in measures of awareness used in studies of unconscious processing. Hence, we concede that the criticism raised by Shanks (2017) is of great importance to the field; without accounting for that problem, one cannot convincingly claim that findings indeed reflect unconscious processing, rather than RttM-based false findings.

How, then, can the field address this crucial point? One option is to avoid post hoc selection altogether. Some avoid excluding participants by only testing objective performance at the group level (e.g., Kouider & Dehaene, 2007; Stein et al., 2021; Van Opstal et al., 2010). Yet with this approach, some of the included participants might be highly aware of the stimuli (e.g., *d′* = 3; Van Opstal et al., 2010). The ideal solution would be to design an experiment where all participants are indeed unaware, so that no exclusion would be needed; however, given the individual differences between participants, this is not easy to accomplish (e.g., Albrecht et al., 2010; Hesselmann et al., 2016; Pessoa et al., 2006). Indeed, in some studies that attempted to do so, some of the participants were above chance when tested for objective visibility (e.g., Stein et al., 2020, 2021). Another possible solution is to tailor the experimental conditions for each participant via calibration (e.g., Hesselmann et al., 2016), as was originally suggested by Shanks (2017), but this approach injects experimental variability, as conditions vary between participants. Furthermore, it does not guarantee a complete lack of awareness, as visibility is likely to increase during the experiment due to practice (Schwiedrzik et al., 2009). While changes in awareness might be addressed by online calibration throughout the experiment, this necessarily means that some of the trials would involve conscious processing (otherwise, no calibration would be needed), which might affect the unconscious trials (Lin & Murray, 2014). And so, avoiding post hoc selection might not be a feasible, practical solution for researchers targeting unconscious processing.

An alternative approach is to correct for the effect of RttM, or develop some statistical tool to test if the results could be explained by it. In fact, such solutions have been suggested by both Shanks and colleagues (Rothkirch et al., 2022; Shanks, 2017; for detailed discussions see sections “Compatibility With RttM solution”, “Split solution”, and “Campbell and Kenny solution” in the Supplementary Materials), and by others (Goldstein et al., 2022; Leganes-Fonteneau et al., 2021); see sections “Generative Bayesian Framework” and “Bayesian Awareness Categorization Technique [BACT]” in the Supplementary Materials). However, as we will show below, these solutions suffer from different limitations and underlying assumptions that render them less suitable for differentiating between genuine effects and RttM-based ones. Specifically, the suggested tests either assume that awareness scores are distributed normally, that their measurement error is independent of their true scores (homoscedasticity), or that they are linearly related to effect scores. The first two assumptions are problematic by definition when applied to objective measures of awareness. The normality assumption may hold if the sample includes only unaware participants, when the true scores are at chance and participants distribute symmetrically due to random measurement error. However, objective performance is expected to distribute asymmetrically if all participants show above chance performance (where the distribution will be truncated at the chance value), or if there is a mixed distribution of aware participants performing above chance, and unaware participants performing at chance level. The homoscedasticity assumption is violated since objective measures are mostly based on alternative forced choice (AFC) tasks. In such tasks, the measurement error is correlated with awareness scores, due to the properties of binomial noise. The third assumption, that the relations between awareness scores and the observed effect is linear, is also problematic. There has not been, to our knowledge, a convincing demonstration that this is indeed the case. Though assuming a linear relationship between these two variables is common, it has not been supported by data. We accordingly argue that under this somewhat ambiguous situation, solutions that do not rely on any such assumption are preferred. This argument is supported by previous works which have already criticized the practice of relying on this assumption of linear relations (or any parametric model) to extrapolate effect scores based on awareness in studies of unconscious processing (Dosher, 1998; Klauer et al., 1998; Merikle & Reingold, 1998; Rouder et al., 2007).

Beyond these potentially problematic assumptions, other solutions suffer from additional limitations. Some of them rely on the reliability of awareness measures. As we show below in the section “Reliability of Awareness Measures”, this reliability tends to be low. Other solutions require that all included participants show evidence for chance level performance (using Bayes factors), which often leads to low power (see section “Testing the Proposed Solution” and section “Bayesian Awareness Categorization Technique [BACT]” in the Supplementary Materials).

Instead, we propose a new method, the *nonparametric bootstrapping solution* (henceforth the NPB solution), which does not rely on any of the above-mentioned assumptions. Our solution provides high specificity and relatively high power for detecting unconscious effects. As we explain in greater detail below, the NPB solution is a multi-phased nonparametric bootstrapping procedure. We first create a null distribution designed to mimic RttM, where no true unconscious effect exists, and the seemingly unconscious effects are completely driven by aware participants who were wrongly classified as unaware due to measurement error. Then, we ask how extreme the observed unconscious effect in the data is with respect to this null distribution.

In this paper, we explain why existing approaches fall short in dealing with RttM, and present our solution, while discussing its advantages and limitations. We describe both simulations and a reanalysis of real data. We collected datasets from 15 different publications,^4^ where 43 unconscious processing effects were tested.^5^ Out of the collected effects, eight effects were not included in any analysis as no participants were excluded (the effects were reported in Van Opstal, Calderon, et al., 2011a; Van Opstal, de Lange, et al., 2011b; Van Opstal et al., 2010; see Supplementary Figure 1), leaving us with 12 studies and 35 effects. Datasets were collected by contacting the first authors of these papers, or using datasets from our lab. We intentionally looked for experiments reporting null results, positive results, or both. This allowed us to assess the reliability of awareness measures, examine the assumptions of the reviewed solutions, and compare their performance with the NPB solution. Before doing so, we will shortly describe the methods we have used for all simulations throughout this paper (with a few exceptions, described in the Supplementary Materials). Readers who are less interested in these more methodological details are welcome to skip to the “Reliability of Awareness Measures” section below.

## General simulations framework

Unless stated otherwise, the simulations reported in this work were performed in the following manner: Using 1,000 iterations, we simulated trial-by-trial awareness and effect scores for each participant according to a combination of several controlled simulation parameters. *Awareness scores* were defined as performance in a 2AFC task and *effect scores* were defined as the difference between two experimental conditions (e.g., trials where a prime and a target are either congruent or incongruent). *True awareness scores* were sampled according to a controlled simulation parameter defining the ratio of truly unaware participants as 0%, 25%, 50%, 75%, or 100% of the entire sample. The true awareness scores of these unaware participants were fixed at chance-level performance (accuracy rate of 0.5). The true awareness scores of aware participants, on the other hand, were sampled from a half-normal distribution (truncated at 0.5, which is the proportion of correct responses for chance level performance) with a standard deviation of 0.15 (expected mean performance of 0.62, and a median lower than 0.6).

Then, we defined the *true effect scores* of the participants. We reasoned that for aware participants, an effect might be driven by both a conscious component and an unconscious component, while for unaware participants, only an unconscious component should drive performance. The conscious component depended on a controlled parameter that defined the type of relation between the true awareness scores and the true effect (linear, exponential, quadratic, logistic or square root). This was done with an upper bound of Cohen’s *d* = 1.2, such that a very large effect was assigned to a fully aware participant, and an intermediate effect was assigned to a participant with intermediate awareness, depending on the simulated relation. The unconscious component was fixed either at Cohen’s *d* = 0 or 0.2, for simulating an absence/existence of an effect, respectively. Then, for aware participants only, the true effect was defined as the sum of the *unconscious* and *the conscious components*.

*Observed awareness scores* were sampled from a binomial distribution with the *number of simulated trials* (a controlled simulation parameter that defines the number of repetitions of the awareness and effect measures for each participant), and the *true awareness scores* of each participant as parameters. *Observed effect scores* were generated by adding gaussian noise to the *true effect scores*.

The number of participants in our simulations was set to 165, corresponding to the required sample size for detecting a small effect (Cohen’s *d* = 0.2), with 80% power (one-sided *t* test, alpha = 0.05; considering an expected rate of 5% false exclusions of participants due to a one-sided binomial test exclusion threshold). All of the code used to conduct these simulations is available at https://osf.io/589nt/.

## Reliability of awareness measures: A possible solution to RttM?

As explained above, RttM is driven by aware participants who are wrongfully considered unaware due to measurement error, leading to an overestimation of the “unconscious” effect (assuming that aware participants show a stronger effect). Thus, estimating the magnitude of the error, or the reliability of awareness measures, is key for estimating the threat by RttM. Potentially, if we could minimize measurement error, thereby increasing reliability, RttM would be minimized—possibly to a point where it would no longer be potent enough to explain the results (Shanks, 2017; but see again Footnote 3). Indeed, some solutions focus on the reliability of awareness measures as means to deal with RttM (Rothkirch et al., 2022; Shanks, 2017; see again the Supplementary Materials).

**Fig. 2:**
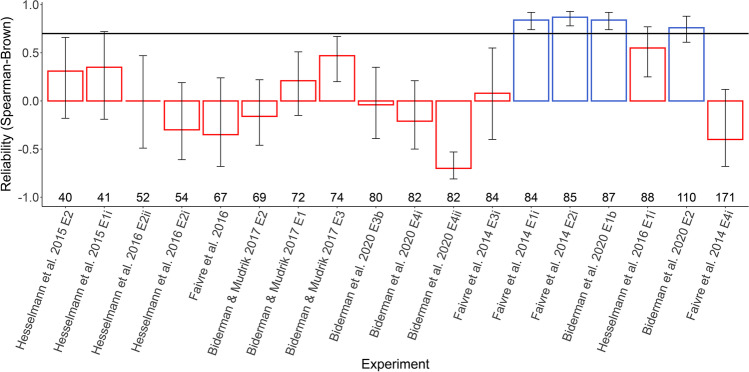
Analysis of reliability for awareness measures in the obtained datasets (*N* = 18; only datasets for which trial-by-trial data was included could be analyzed). Reliability was calculated using a split-half reliability test with Spearman–Brown correction across 5,000 random splits. Error bars depict the bootstrapped 95% CIs according to all random splits for each dataset. Numbers over the *x*-axis denote the average number of trials in the awareness measure. Red and blue bars denote reliability measures below and above the acceptable reliability threshold we set (0.7). (Color figure online)

To assess the scope of the reliability problem in the data we obtained, we first analyzed the Spearman–Brown corrected reliability of awareness measures (estimated across 5,000 random splits) in all studies in which the trial-by-trial data was available﻿ (*N* = 18 experiments; Fig. 2). Adopting a threshold of 0.7 (Cortina, 1993), only four experiments were found to have reliable awareness measures (note that for two additional experiments in Sklar et al., 2012, no trial-by-trial data was not available. Thus, reliability was not analyzed here. Notably, they reported high reliabilities of .93 and .83, with 64 trials), with the others showed relatively low, and even negative, values (for similar results see Rothkirch et al., 2022, where the reliability of awareness measures used in implicit learning and unconscious processing studies was assessed).

On the face of it, this seems like a major blow to the field; if the awareness measures used to demonstrate a lack of awareness are unreliable, how can we convincingly claim that any of our effects indeed represent unconscious processing? The answer to this question lies, in our opinion, in the expected pattern of results if participants are truly unaware, which—as we claim below—should by definition lead to low reliability scores, even if the measure itself is in fact accurate. To understand that, we should first dive deeper into the meaning of reliability in this context. As a reminder, split-half reliability is calculated by iteratively dividing the data of each participant into two halves, and calculating the correlation between the two sets. In order for such correlations to be found, leading to high reliability, *individuals should systematically differ in the measured features*. That is, the variability in these measures must be a meaningful one (Hedge et al., 2018; Spearman, 1910). Typically, for very noisy measures, low reliability will be driven by high measurement error that masks the true scores. Yet, in the case of unconscious processing, *low reliability might actually stem from the true scores rather than from measurement error*. Remember that if participants are truly unaware, their true awareness score should be at chance level. And so, there should be no *meaningful* variation in the true scores that the measure could capture (see Footnote 8 in Rothkirch et al., 2022, for a similar argument). Thus, the only thing that would differentiate between individuals is the *random* measurement error (see again Fig. 1). In that case, no correlations should be found, because the measurement error, being random, will not covary systematically between the halves. Accordingly, *the more truly unaware participants in the sample, the less reliable the measure will be*. Interestingly, a similar result was obtained outside the field of unconscious processing, when examining some of the most robust effects in cognitive science (e.g., Stroop, go/no-go, SNARC, or flanker tasks). There, although the effects are very robust, the reliability is low. This apparent paradox was explained in the same way described here: since the variability between individuals is low for very strong effects, correlational tools cannot serve as good indicators for reliability (Hedge et al., 2018).

To test if this explanation could indeed account for the low reliability scores that we found, we ran a simulation that diverged from the general simulations framework described above, being only focused on awareness scores (and not the effect scores) since our goal was to examine the issue of reliability (see Supplementary section “Reliability Simulation” for more details). We manipulated two factors: first, the number of trials in the awareness measure. This factor was chosen because the number of observations is known to affect reliability (see Supplementary Materials D in Hedge et al., 2018, for an analysis across different cognitive tasks), and this claim has specifically been raised in the field of unconscious processing (Meyen et al., 2022; Shanks, 2017; Vadillo et al., 2022). Thus, this served as a sanity check for our simulation, expecting to find higher reliability when more trials were used. Second, we manipulated the percent of truly unaware participants in the sample, to examine the extent to which variability in true awareness scores is needed for reliability (Hedge et al., 2018); if our argument above is correct, we should expect lower reliability the more truly unaware participants are included in the sample.

**Fig. 3:**
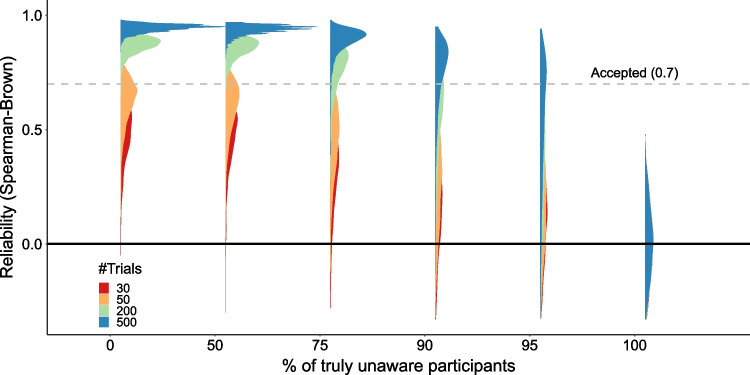
Reliability simulation results across different samples, varying in the percentage of truly unaware participants (*x*-axis), and the number of trials (*N* = 30 [red], 50 [orange], 200 [green], and 500 [blue]). On the *y*-axis, the scores of Spearman–Brown-corrected reliability, for which the distribution across interactions appears for each sample type

Both these expectations were borne out by the simulated data. We found that the number of trials affects reliability, so that the more trials one has, the higher the reliability and the narrower the distribution across iterations (Fig. 3). In line with our argument above, the only case where this did not happen is when the entire sample was composed of truly unaware participants, where reliability is distributed around zero for all trial numbers, and never crosses the 0.7 threshold. And most importantly, low reliability was also found when there were 90% or even 75% unaware participants, especially when the number of trials was low (that is, the more the sample includes above-chance participants, the higher the reliability). Thus, with the same awareness measure, reliability dropped as a function of the percent of truly unaware participants, showing that it is the latter factor, rather than the reliability of the measure itself, that drives the observed low reliability scores (calculated using the Spearman–Brown method).

**Fig. 4:**
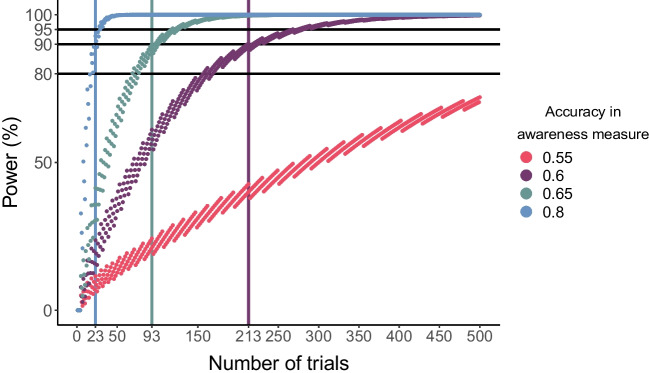
Binomial test power estimation. The *y*-axis depicts the probability of detecting an aware participant given the number of trials (*x*-axis) and the accuracy scores where chance is 0.5 (accuracy = 0.55 [pink], 0.6 [purple], 0.65 [green], and 0.8 [blue]). Horizontal lines represent accepted power thresholds (0.8, 0.9, 0.95). Vertical lines represent the threshold for 90% power. (Color figure online)

Focusing on samples with more aware participants allows us to further stress the point about the contribution of the number of trials to correctly assessing participants’ performance (Meyen et al., 2022; Shanks, 2017; Vadillo et al., 2022). In these samples, between-participants variability is meaningful (as true scores are not at chance), yet with a low number of trials, reliability is lower than 0.7. To further highlight the importance of the number of trials, we examined how it affects the power of binomial tests (which are often used as means to exclude participants; e.g., Rothkirch et al., 2015; Sklar et al., 2012) to detect an aware participant. We systematically manipulated the number of trials from 1 to 500 for a given aware participant taking a 2AFC awareness task (with varying accuracy levels: *p* = .55, .6, .65, and .8) and calculated the power of a binomial test to detect above-chance awareness. As Fig. 4 shows, when accuracy is high (0.8), even 23 trials suffice for 90% power. However, when accuracy is closer to chance, as is often the case in studying unconscious processing, a much larger number of trials is needed (for 0.6, 213 and for 0.55, 866). Notably, participants with such low accuracy are at a greater risk of being misclassified as unconscious (thus contaminating the group-level effect with conscious processing).

Taken together, two important conclusions should be drawn from the results of the current section. First, experimenters should assign a large enough number of trials for the awareness measures, especially when conducting a binomial test to screen out participants. Second, even with sufficient trials, as we explained above, it will be hard to draw any conclusions about measurement error from the obtained reliability of their measures, especially if most participants are truly unaware. Thus, assessing the reliability of awareness measures (e.g., using Spearman–Brown) is not expected to yield meaningful insights about the reliability of the awareness measures, as reliability is *expected to be low exactly when the awareness manipulation is effective*. Finally, given the latter conclusion, current approaches which rely on assessing reliability simply do not provide a good enough solution for RttM.

## The proposed solution (NPB)

We are proposing a nonparametric solution for addressing RttM. The basic idea is to use resampling methods to create surrogate datasets with no effect of interest (no unconscious processing) while maintaining other properties of the original data, including the potential effect evoked by RttM. We then compare our observed effect to the distribution of effects estimated from surrogate data to determine whether RttM could explain our observation in the absence of unconscious processing.

To motivate our method, we want to highlight two main approaches for dealing with the RttM problem, focusing on the exclusion threshold. The usual situation is that participants are excluded from the analysis based on some threshold (so that only participants whose awareness scores are lower than that threshold are included; we will call this threshold the *initial threshold*). The impact of RttM on the group-level effect is driven by the misclassification of aware participants as unaware (truly aware participants, who randomly fall below the exclusion threshold due to measurement error). One potential approach is to lower the exclusion threshold (i.e., choose a *conservative threshold*), thereby reducing the potential for misclassified participants, and thus the magnitude of RttM-driven effects. The drawback of this approach is that it results in misclassifications in the other direction (truly unaware participants classified as aware), reducing the number of participants used in the analysis and thus the power of the statistical test. The second potential approach is to keep the initial threshold, and attempt to estimate the effect of RttM. As mentioned above, however, estimates of the effect of RttM are usually based on some assumptions about the data (normality, linearity, etc.), which are not always justified.

**Fig. 5:**
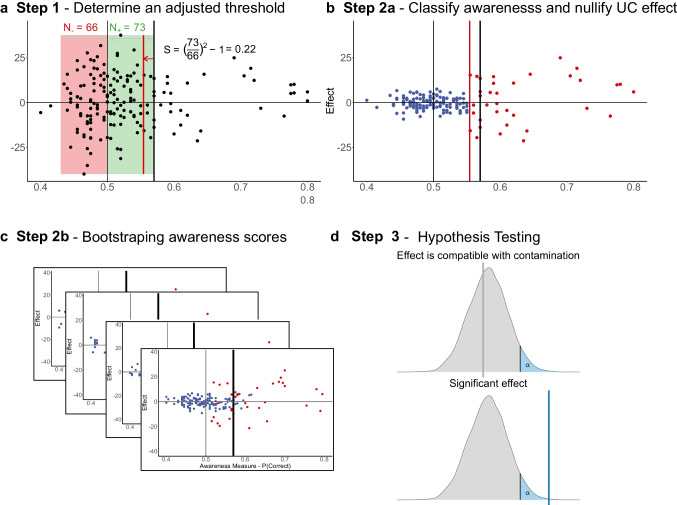
Demonstration of the steps of the proposed solution. Panel **a**: the negative and positive halves of the expected unaware scores according to the *initial threshold* (indicated by the black vertical line) are highlighted in red and green, respectively. The number of participants in each half is indicated above. The *adjusted threshold* (*h*_*adj*_), indexed by the red vertical line, is set according to the asymmetry measure *S*; Panel **b**: blue and red dots are scores of participants that were classified as unaware and aware, respectively, according to the *adjusted threshold*. A permutations procedure nullifies the effect scores of the former group while the latter scores keep their original values; Panel **c**: bootstrapped awareness scores in different iterations, resulting in different participants being included in the calculation of the effects, defined by the *initial threshold* (*h*), mimicking the effect of RttM and conscious effects contamination; Panel **d**: an illustration of the resulting distributions of RttM driven effects, compared with the observed effect. Upper panel: the observed effect is not larger than 95% of the values in the distribution of RttM-driven effects; Lower panel: similar to the upper panel, except that the observed effect is significantly greater than 95% of the RttM mimicked distribution. (Color figure online)

Our proposed solution uses resampling methods and combines these two approaches without making any assumptions about the data. Starting with the *initial threshold* (e.g., the critical value of a binomial test or some fixed threshold set by the researcher, like 0.65) we compute an observed effect. We then estimate an adjusted (*conservative*) threshold for which participants below the threshold are almost certainly unaware. Participants below this adjusted threshold are then subjected to a permutation procedure to create surrogate data with no unconscious processing. Next, participants above the adjusted threshold are used to create a bootstrap sample of potentially aware participants that can be used to estimate the distribution of RttM-driven effects. Repeating this process many times allows us to estimate an empirical distribution of RttM-driven effects in the absence of unconscious processing. If the observed effect lies outside a 95% confidence interval for the mean effect evoked by RttM (Fig. 5), we conclude that the effect cannot be explained by conscious processing and RttM.

### Step 1: Determine an adjusted threshold

As the impact of RttM on the group-level effect is driven by the misclassification of aware participants as unaware, the solution first uses an exclusion threshold *h* (the *initial threshold*). In our simulations, we set the *initial threshold* to be the 97.5% percentile of a binomial distribution (with *p* = .5 and *n* = the average number of trials used to assess awareness; henceforth, the “*chance distribution*”), which is also the critical value of a two-sided binomial test of the hypothesis *p* > .5. We generally recommend that this method will be used to set the initial threshold, as this method was validated in our study.

The primary goal of Step 1 is to derive an *adjusted threshold* for which participants below the threshold are almost certainly unaware. To determine if, and to what extent, the threshold should be adjusted, we compare the number of included participants with awareness scores lower than chance (i.e., located at the negative half of a binomial distribution around chance-level performance) with the number of included participants with awareness scores above chance (i.e., located at the positive half of the distribution) given that the true awareness score of such participants is 0.5 (chance level performance), and that binomial noise is symmetric around zero, we should expect an equal number of truly unaware participants who are above and below chance. Thus, if we find asymmetry in the distribution of awareness scores around chance, this implies that some of these participants are actually aware. To put it explicitly, we assume that any deviation from symmetry in the distribution of awareness scores around chance level (i.e., when more participants are showing above chance performance than participants showing below chance performance) represents contamination by conscious participants, and requires the adjustment of the exclusion criterion.

Using this symmetry assumption, we quantify a measure of the potential contamination by aware participants. This measure will be later used for adjusting the *initial threshold*. To do so, we define *δ* = *h* − 0.5 to be the distance between the *initial threshold* and chance performance, used to then define equal-sized negative [0.5 − *δ*, 0.5) and positive [0.5, 0.5 + *δ*] intervals on either side of chance, and count the number of participants in each interval (with the exact chance point belonging to the positive half). We subtract 1 from the squared ratio of these numbers to accentuate any deviance from symmetry while keeping a value of 0 for a completely symmetrical distribution. Then, we define a measure of asymmetry *S* (Eq. 1; *N*_+_ and *N*_−_ denote the number of participants in the positive and negative intervals of awareness scores):

1 $$S={\left(\frac{N_{+}}{N_{-}}\right)}^2-1$$

Next, we use S to define an *adjusted threshold* (*h*_*adj*_). This new threshold is intentionally more conservative than the *initial threshold*, with the adjustment being a function of the potential contamination by conscious processing assessed above using S. With the adjusted threshold, included participants should (almost certainly) be unaware. If the asymmetry measure *S* is negative (i.e., there are more participants in the negative half than in the positive half of the chance distribution), or is equal to zero, we set the *adjusted threshold* equal to the initial threshold. Otherwise, we decrease *h* by weighting the standard deviation of awareness scores, which is critical for the sensitivity of awareness tests (see again Fig. 4), by the asymmetry measure *S*, according to the following formula:

2 $${h}_{adj}=h-S\frac{2}{\sqrt{4n}}$$

Again, *h* denotes the *initial threshold*, *S* is the asymmetry measure derived from Eq. 1, and $\frac{2}{\sqrt{4n}}$ is two standard deviations (*SD*s) of the average awareness score under the chance distribution (indexing the expected binomial variability in awareness scores). Thus, the *adjusted threshold* is also a function of *n*, representing the number of trials (since the greater the number of trials, the smaller the standard deviation, and accordingly—the less substantial the adjustment of the threshold). Our choice of adjustment to the threshold is a heuristic method, and it is worth noting that other possible solutions might achieve the goal of separating unaware and potentially aware participants in a sufficiently conservative manner.

### Step 2: Create surrogate data that can be used to estimate the null distribution of RttM

As a reminder, under the null hypothesis, there is no unconscious effect (but there might be a conscious one). And so, if aware participants are misclassified as unaware due to measurement error, they might contribute to falsely finding an unconscious effect. To create surrogate data that can be used to estimate this contamination (i.e., the impact of RttM) under the null hypothesis we need to emulate these two aspects: define truly unaware participants who show no effect, and potentially aware participants who might show it, and simulate new random measurement error that can lead to different patterns of misclassification, and thus contamination by conscious processing due to RttM.

### Step 2A: Define truly unaware and potentially aware participants, and nullify the effect of true unaware participants

To create surrogate data under the null hypothesis, we consider participants whose awareness score is below (or equal to) the *adjusted threshold h*_*adj*_, as truly unaware, and participants whose awareness score is above the threshold, as potentially aware. To ensure the surrogate data follow the null hypothesis, for each truly unaware participant, we nullify the effect scores by permuting the condition labels of each trial. The permutation procedure results in an average effect score of zero, while maintaining other marginal properties of the data. No manipulation is performed on the aware participants, so their effect scores remain the same (Supplementary Eq. 1).

### Step 2B: Bootstrap awareness scores, to simulate the misclassification of aware participants as unaware

Following Step 2A, we have unaware participants who show no effect and aware participants who show an effect. When some of these aware participants would be misclassified as unaware, the group-level effect is contaminated by conscious processing due to RttM. To simulate this scenario, we manipulate the observed awareness scores by resampling awareness scores from a binomial distribution based on the number of trials used to measure awareness (so that bootstrapped awareness scores have more variability when there are fewer trials ). Thus, for each participant, we randomly sample an awareness score from a binomial distribution (with *p* = the observed awareness score, and *n* = the number of trials used to assess awareness for each participant; Supplementary Eq. 2). Accordingly, the distribution of the *bootstrapped awareness score*s, across iterations, is still centered around the original observed awareness scores, yet are different from their observed scores. That is, by chance some unaware participants who show no effect—as it was nullified—will have awareness scores higher than the threshold. Yet crucially for estimating the effect of RttM, some aware participants who showed awareness scores above *h*, whose effect had *not* been nullified, will now have awareness scores below *h*. As we explain in the next step, these bootstrapped awareness scores can now be used to estimate the distribution of RttM-driven effects due to the misclassification of some of the aware participants as unaware.

Note that Steps 2A and 2B can be performed in any order. That is, after defining the *adjusted threshold* (*h*_*adj*_), nullifying the effect of unaware participants via permutation and bootstrapping the awareness scores of potentially aware participants are two independent steps.

### Step 3: Calculate the effect of RttM in surrogate data and estimate an empirical distribution

For each surrogate dataset, we use the *initial threshold* (*h*) and the bootstrapped awareness scores to define the included and excluded participants for this surrogate dataset (Supplementary Equation 3). The rationale is simple: we use the *initial threshold* to determine which participants to include for surrogate datasets, following the same cutoff of the originally calculated effect. Notably, in each iteration different participants are included, in a manner that takes measurement error into account (since the bootstrapped samples are based on the observed data and the number of trials): some of the participants in the included sample belong to the *truly unaware* sample defined above (participants with awareness scores below *h*_*adj*_), for which the permutations nullified the effect, while other participants belong to the *potentially aware* sample, for which the effect did not change. This mimics the RttM threat described by Shanks, whereby aware participants are misclassified as unaware, potentially driving the observed effect even when no effect exists for truly unaware participants. Hence, when we then calculate the average of the surrogate bootstrapped effect scores in the included group (Supplementary Equation 4), we obtain the group-level *RttM-driven effect* for the surrogate data in that iteration. By iterating this procedure many times (here, we perform 1,000 iterations), we estimate the null distribution of RttM-driven effects, to which the originally observed effect (calculated according to the observed awareness, effect scores, and the *initial threshold*; Supplementary Equations 5–6), is then compared. If the effect is greater than the 95th percentile, we deem it non-RttM evoked, and—under this framework—significant (Supplementary Equation 7).

## Testing the proposed solution

**Fig. 6:**
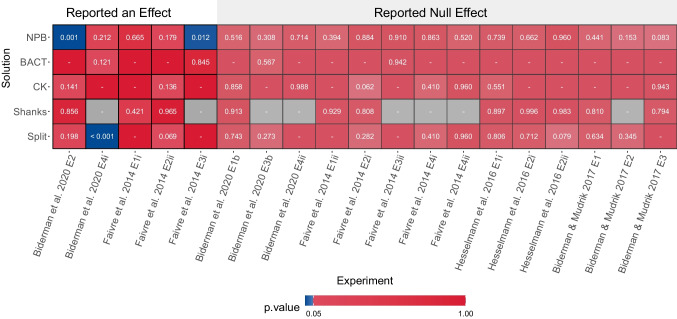
Results of applying the different solutions on the acquired datasets (*N* = 19; only datasets that used 2-AFC tasks to measure awareness and included trial-by-trial data were analyzed). The *x*-axis labels denote the name of the tested effect, with experiments that reported positive results on the left and null results on the right. The *y*-axis labels denote the different solutions tested here. Each cell reports the *p*-value obtained for each effect by each solution. Blue cells denote significant results (alpha = .05), red cells denote non-significant results (when the solution did not find enough evidence for unawareness in the sample to assess the significance of effects were marked with “-”), and gray cells indicate that the Shanks solution could not be applied because no participants were excluded from the analysis post hoc based on awareness measures. Abbreviations: NPB = the proposed solution; BACT = Bayesian awareness categorization technique (Leganes-Fonteneau et al., 2021); CK = Campbell and Kenny's solution (Campbell & Kenny, 1999; Rothkirch et al., 2022); Shanks = RttM compatibility solution (Shanks, 2017; see section “Compatibility With RttM” in the Supplementary Materials where we question the validity of this solution); Split = relying on consistent BF based group-level demonstration of chance level performance (Shanks, 2017). As the figure shows, all solutions were conservative. Yet as opposed to the NPB solution, other solutions classified too few participants as unaware to even assess the significance of effects (BACT: 15 datasets, CK: 10 datasets, Split: six datasets, and Shanks: eight datasets; see also Supplementary Figure 3 where we show that Shanks test actually tests for deviations from linearity, and Supplementary Figure 5 where we examine the number of participants included in the BACT). All but the Split solution (which found one reliable effect) did not mark any of the reported positive effects as reliable, contrary to the NPB solution which found two reliable effects. Finally, the Shanks solution did not find any positive results (yet see again Supplementary Figure 3). (Color figure online)

First, we used our solution to examine the datasets we acquired, using the original exclusion threshold on awareness measures used by the researchers in each experiment as the *initial threshold* (*h*). We further compared the results of our solution with the alternative solutions suggested in the literature—namely, the BACT (Leganes-Fonteneau et al., 2021), the Campbell and Kenny solution (Campbell & Kenny, 1999; Rothkirch et al., 2022), the Shanks solution and the Split solution (Shanks, 2017). The results yielded a mixed picture. According to the Shanks solution, the Campbell and Kenny (CK) solution, and the BACT, none of the effects proved genuine after accounting for RttM (Fig. 6; see also section “Bayesian Awareness Categorization technique [BACT]” in the Supplementary Materials for an additional analysis of the performance of this solution with a more liberal prior^6^). Furthermore, according to the BACT, in 15 out of the 19 datasets, only one (*N* = 9) or no (*N* = 6) participants were considered unaware, not allowing us to test for an unconscious effect. Similarly, the CK and Split solutions excluded all participants in ten and six of the datasets, respectively. In contrast, other solutions showed various positive results. Specifically, our solution revealed that two out of the five reported significant effects were reliable, and the “Split” solution found a single such effect, while for all solutions none of the experiments reporting null effects yielded positive results. However, the ability to draw conclusions from real data is limited, as we do not know the ground truth. This relates mainly to the existence/absence of an effect in these datasets, as well as to the number of unaware participants, which was found by some of the solutions to be extremely low (too low to test for unconscious effects) in many of the datasets. For these datasets, we simply do not know if this means the tests are too harsh, misclassifying unaware participants as aware, or if these participants were indeed aware, and existing tests are not sensitive enough to detect that. To provide a stronger test for the different solutions, we used simulations (see the General Simulations Framework Section).

**Fig. 7:**
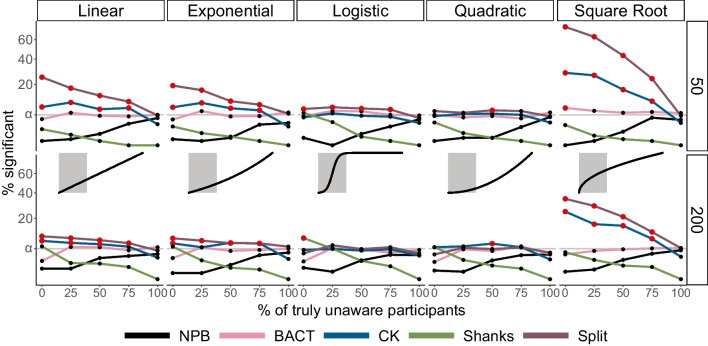
Using simulations to examine the false-positive rate of the suggested solutions. For all simulations, no unconscious effect exists. Black, pink, blue, green and purple lines denote the percentage of significant effects found by the NPB, BACT, CK, Shanks, and Split solutions, under different simulation parameters combinations, manipulating the relations between awareness and effects (vertical panels; insets depict the true relations between awareness and effects, with the area highlighted in gray corresponding to true awareness scores of 50–100%; see Supplementary Table 1 for the formulas used for each relation), percentage of truly unaware participants (*x*-axis) and the number of trials used to measure awareness (horizontal panels). Red colored points indicate that the proportion of false-positive effects exceeds *α* = 0.05 signalling an inflated false-positive rate (higher than the upper bound of a 95% confidence interval around *α*=5% according to a random process with *p* = .05). The number of participants was set to 165 in all of the tested conditions. As can be seen, NPB is the only one not exceeding 5% under any scenario. The BACT solution also provides relatively high specificity, yet the other solutions do not (although the results may hint that Shanks' solution shows relatively high specificity, in Supplementary Figure 3 we show that this is not the case with powered samples and more crucially, when its assumptions are met, revealing that the test in fact tests for deviations from linearity and not for RttM). A square root scale was used for the *y*-axis to facilitate comparing the solutions around the *α* = 0.05 threshold requirement. (Color figure online)

The simulations examined the rate of significant unconscious effects using one-sided tests, because the sample size was determined according to an expected 80% power in a one-sided *t* test (except for the Shanks method which was examined against a 95% confidence interval around the expected effect, see section “Compatibility With RttM Solution” in the Supplementary Materials), in different conditions. We manipulated three factors: the type of relations between awareness and effect scores, the number of truly unaware participants, and the number of trials. The results show that our solution keeps the key requirement for statistical tests, controlling the false-positive rate (e.g., Lakens et al., 2020) for all combinations of these factors (Fig. 7). Given ongoing methodological criticisms and general skepticism towards the findings in the field, we suggest that high specificity (here using the standard alpha of 5%) is especially important when testing for unconscious processing effects. As a reminder, the entire issue of RttM is focused on the concern that a null effect will be interpreted as a true one. Our solution mitigates that concern. Relatively high specificity was also found for the BACT (note that although the results may imply specificity also of the Shanks solution, this is somewhat misleading since we show that this solution suffers from a more crucial issue of validity, testing for deviation from linearity rather than unconscious effects; see Supplementary Figure 3). In contrast, the CK and Split solutions showed an inflated false-positive rate, under different conditions, which in some cases was higher than 28% and even 75%.

**Fig. 8:**
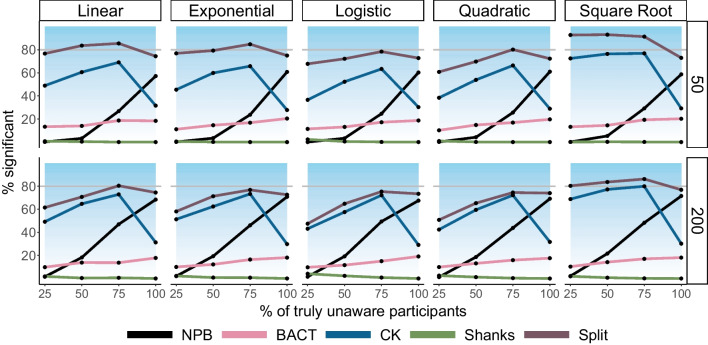
Using simulations to examine the power of the suggested solutions, so that here, in all simulations an unconscious effect exists (Cohen’s *d* = 0.2). The horizontal line at 80% indicates the power of a one-sided *t* test for detecting an effect of truly unaware participants assuming an exclusion rate of 5%. As in Fig. 7, each line denotes the percent of significant effects found under different simulation parameter combinations for each solution. The blue background color represents increasing power. As can be seen, the power of our solution grows with more unaware participants in the sample and with more trials. The Bayesian solution yields very low power (see Supplementary Figure 5 for the number of included participants in each cell), and the Shanks solution misses true effects in the vast majority of the cases, across all conditions. The Split and CK solutions provide higher power, yet as Fig. 7 shows, their false-positive rate is also high. Note also the effect of increasing the number of unaware participants on their power, and the unexpected decrease of power for the Split method with more trials (e.g., when comparing the results of the solution in the Square Root condition).

Yet specificity is not enough; if the solution is not sensitive, it will not be a viable solution. Our simulations suggest that sensitivity was strongly modulated by the number of trials, and the percentage of truly unaware participants. When all participants are unaware, and with 200 trials, the power of our solution ranges between 67.5% and 71.5% (Fig. 8). Similar results are obtained irrespective of the simulated relations between the effect and awareness scores, demonstrating that our solution is robust under different relations between these variables which is important given the lack of knowledge about these relations, as we explain above.

Importantly, though, the more participants are aware, and with a lower number of trials, the power of our solution decreases—which again can be expected. This shows the sensitivity of the proposed solution to the latent parameter of the ratio of truly unaware participants, which has direct consequences for the potential for contamination by conscious processing (see section “Erroneously Inferring Unconscious Processing From Fully Conscious Samples” in the Supplementary Materials, showing an additional condition where this feature is crucial to differentiate between conscious and unconscious processing effects). In a way, this is a feature and not a bug: If there are too many conscious participants in the sample, our solution will justly show reduced chances for declaring an unconscious effect (as the data is more heavily affected by conscious participants, and the threat for contamination by conscious processing is greater). In contrast to the proposed solution, the BACT solution, the only alternative solution that seemed to provide good enough specificity with regard to detecting true unconscious effects, showed drastically lower sensitivity (ranging between 17.6% and 19.1% when all participants were truly unaware, and the number of trials used to assess awareness was 200). The lack of sensitivity in this case is directly related to the high bar the BACT solution sets for classifying participants as unaware, thereby reducing the effective sample size (see Supplementary Figure 5 for the relation between the true number of unaware participants and the included sample for the simulated conditions). The other solutions sometimes showed higher sensitivity (e.g., 93.1% for the Split solution with square root relations and 50 trials). This higher sensitivity was further demonstrated in an Area Under the Curve (AUC) analysis, which incorporates both specificity and sensitivity together. Yet, given the high false-positive rate this seems less meaningful (e.g., the Split solution shows the highest AUC amongst all solutions with Square Root relations and 50 trials due to very high sensitivity, but at the cost of a highly inflated false-positive rate of up to 75.6%; see Supplementary section “Comparing the Solutions Using an Area Under The Curve [AUC] Analysis” for all results). Also, for both the Split and the CK solutions power decreases when more participants are unaware (e.g., 73% and 29.2% when all are unaware in this case, for the two solutions respectively).

Taken together, our simulations suggest that our proposed solution provides the best combination between sensitivity and specificity, by controlling the false-positive rate while allowing for relatively high power to detect effects, especially with more unaware participants in the sample. This property is maintained irrespective of the relations between awareness measures and effects, in contrast to other solutions that may perform well under some conditions (e.g., the Logistic relation; see Figs. 7 and 8), yet compromise specificity. The reduced specificity is especially prominent when aware participants with low awareness scores already show detectable effects (e.g., in the Square Root relation, Fig. 8, and section “Erroneously Inferring Unconscious Processing From Fully Conscious Samples” in the Supplementary Materials). We accordingly suggest that NPB is a relatively strong tool for examining whether an observed effect might be driven by regression to the mean or indeed reflects unconscious processing.

## Limitations of the proposed solution

Researchers adopting our solution should take into account its limitations. First, our solution is nonparametric, and its power is accordingly lower than that of an appropriate parametric test. This was also evident in the sensitivity analyses reported above. However, we think that the choice of a nonparametric test is preferable given the ambiguity around the relation between awareness level and the effect. Moreover, because the field currently suffers from some degree of uncertainty (Peters et al., 2017; Rothkirch & Hesselmann, 2017; Shanks et al., 2021, p. 20), it seems safer to adopt a stricter approach and refrain from making potentially unjustified assumptions, in an attempt to establish a more reliable and replicable science of unconscious processing. Hence, we argue that the cost of a somewhat lower sensitivity is worth the benefit of establishing firmer grounds for claims about unconscious processing.

Second, like other solutions, NPB relies on the awareness measure scores for assessing RttM. We assert that our solution better accounts for reliability issues than the other ones because it does not expect awareness scores to show consistent variability. Instead, our solution assumes that if participants are indeed unaware, their awareness scores—by definition—should be randomly distributed around chance. Indeed, our simulations show that our solution is more sensitive than the others when a relatively low number of trials is used to measure awareness, implying lower reliability (Fig. 3). However, this raises additional issues. Objective measures of the type we used here have been criticized for being overly restrictive (or overestimating awareness), since above-chance performance might index unconscious processes rather than conscious ones (Cheesman & Merikle, 1986; Reingold & Merikle, 1988). In addition, there is an ongoing discussion about the appropriate level for the objective measure (i.e., should it pertain to having any information about the stimulus, or to the feature of interest only; Michel, 2022). Thus, if one chooses an inappropriate awareness measure, our solution might deem the effect as non-RttM driven although participants could still be aware. For example, if the task is too difficult, participants might be at chance even when aware. The same problem would arise when using a measure with low construct validity, in the sense that it does not measure awareness exclusively or exhaustively (Reingold & Merikle, 1988), see again Footnote 3. Thus, interpreting the results of our solution must always be done with a careful estimation of the appropriateness of the objective measure.

Third, as opposed to other solutions (e.g., Goldstein et al., 2022; Leganes-Fonteneau et al., 2021), our solution cannot be used to determine if an individual participant was aware of the stimuli. Instead, it assesses if the group-level effect can be explained by RttM or not. Fourth, there could be some extreme circumstances in which the NPB solution will yield a false positive rate above the 5% level. For example, this could happen if the true relation between awareness and effects is such that participants who are slightly above chance show a very strong effect and participants at chance show no effect. Notably, the performance of the test in the Square Root relation suggests that our solution has a lower false positive rate, within the expected 5% bounds, compared with the others who pass those bounds in this condition (see again section “Erroneously Inferring Unconscious Processing From Fully Conscious Samples” in the Supplementary Materials, for an example of the results of the solutions on one such condition). Fifth, although our simulations were fairly extensive, including five different types of relations between awareness and the unconscious effect, this is not an exhaustive examination—there could be many more other relations that could have been probed. Future studies might develop other simulations, or provide analytic solutions to test the proposed solution further and potentially improve it.

## Conclusions

In this paper, we tried to address a highly important criticism that was raised against findings of unconscious processing: the claim that they can be driven by RttM (Shanks, 2017). We agree that this is a major problem, and have provided evidence for the underlying mechanism driving this threat—the low reliability of awareness measures. We accordingly suggest a new solution that mimics the possible effects of RttM, as means to determine if a given effect can be explained by it. Our solution goes beyond previous ones in being nonparametric, hence not making any assumptions on the relation between awareness and the effect, and in providing greater power to detect an effect if it exists while keeping a low false-positive rate across different conditions. Importantly, though this solution was developed with studies of unconscious processing in mind, it might prove useful to other fields of research that face a similar threat by RttM.

### Supplementary Information

ESM 1 (DOCX 2.06 mb)

## Footnotes

1 We focus here on objective measures of awareness, as the original criticism was directed at them, and they are indeed widely used. However, some researchers opt for only using subjective measures (Avneon & Lamy, 2018; Sandberg et al., 2010; Sergent et al., 2005), and often exclude *trials* where participants report being aware of the stimuli. Shanks (2017) argued that the same criticism should hold for trial exclusion (see also Schmidt, 2015), but this has yet to be systematically demonstrated in the same way that was done for excluding participants based on objective measures. We accordingly set this question aside for now.

2 And, thus, the lower the correlation, as reliability sets the upper limit for correlation (Weir, 2005).

3 Notably, apart from measurement error, confounding variables can also reduce the correlations between variables, and lead to RttM. For example, if there is a third variable that is not measured and affects performance. In that case, awareness measurements per se could be perfectly reliable, but fail to estimate the contribution of that third variable, and RttM could still occur.

4 Biderman et al. (2020); Biderman and Mudrik (2017); Faivre et al. (2014, 2015); Hesselmann et al. (2015, 2016); Karpinski et al. (2019); Nakashima and Sugita (2018); Reber and Henke (2012); Reber et al. (2014); Sklar et al. (2012); van Gaal et al. (2014); Van Opstal, Calderon, et al., 2011a; Van Opstal, de Lange, et al., 2011b; Van Opstal et al., 2010. See also Supplementary Figure 1 for all of the relations between awareness and effect scores in each experiment. Importantly, datasets for which we had trial-by-trial data were reanalyzed to fit the purposes of this work. Specifically, we targeted main effects which were calculated as difference scores between two conditions. Thus, the effects inspected here may differ from those reported in the original paper.

5 Note that we only focused on studies where the stimuli were rendered invisible using some psychophysical manipulation. This is different from studies of implicit processing, analyzed in Vadillo et al. (2022), where the stimuli are all consciously perceived, but the participants are unaware of the process they perform on it (e.g., learning the governing rule or extracting some relevant information; Seger, 1994).

6 There, one positive effects are found. Notably, however, later we show that this increase in positive results is also found when using this prior when no true unconscious effects exist (see Supplementary Figure 4 and Supplementary Figure 6).
