# Supplementary material for: Progressing, not regressing: A possible solution to the problem of regression to the mean in unconscious processing studies
Source: Psychon Bull Rev. 2023 Aug 1;31(1):49–64. doi: 10.3758/s13423-023-02326-x (PMC10867080; doi:10.3758/s13423-023-02326-x)
Supplement: Supplementary file 1 — (DOCX 2.06 mb) [file 13423_2023_2326_MOESM1_ESM.docx]

**Supplementary Material**

**Progressing, not regressing: a possible solution to the problem of regression to the mean in unconscious processing studies**

**Supplementary Sections**

The supplementary materials include several sections. First, we present the relations between awareness and effect scores in all of the datasets we collected. In the second, we examine existing solutions to the RttM problem. In the third section, we provide the full details of the reliability simulation, used to generate Figure 3. In the fourth, we report the performance of the solutions when testing for unconscious effects based on fully aware samples with potential unconscious effects. In the fifth section, we present an area under the curve (AUC) analysis to compare the performance of the solutions. Lastly, in the sixth section, we provide equations underlying the proposed solution.

**1. Relations between awareness and effect scores in empirical data**

The figure below depicts the relations between awareness and effect scores in the different datasets we acquired (N = 43). All datasets were collected in experiments examining unconscious processing, with the vast majority focusing on the visual modality (see Faivre et al. 2016, for the single exception where multimodal processing between tactile and visual information was explored).

Also, note that we encountered a few small mismatches when reanalyzing the shared data of some datasets: In Hesselmann et al., 2015 1^st^ experiment, the large numerosities effect (labeled Hesselmann et al., 2014 1i in this paper), we could not reproduce the reported reaction times. Furthermore, in Van Gaal et al., 2014, experiment 2, the authors now consider the originally excluded subjects as included. Although we were made aware of that, for the purposes of this paper, we followed the original report as it appears in the paper. Lastly, in (Faivre et al., 2014) our analysis deviates from the reported effects for experiment 4, with both group-level effects being similar in magnitude to those reported in the paper, yet their sign is inverted (labeled Faivre, et al., 2014, 4i, and 4ii).


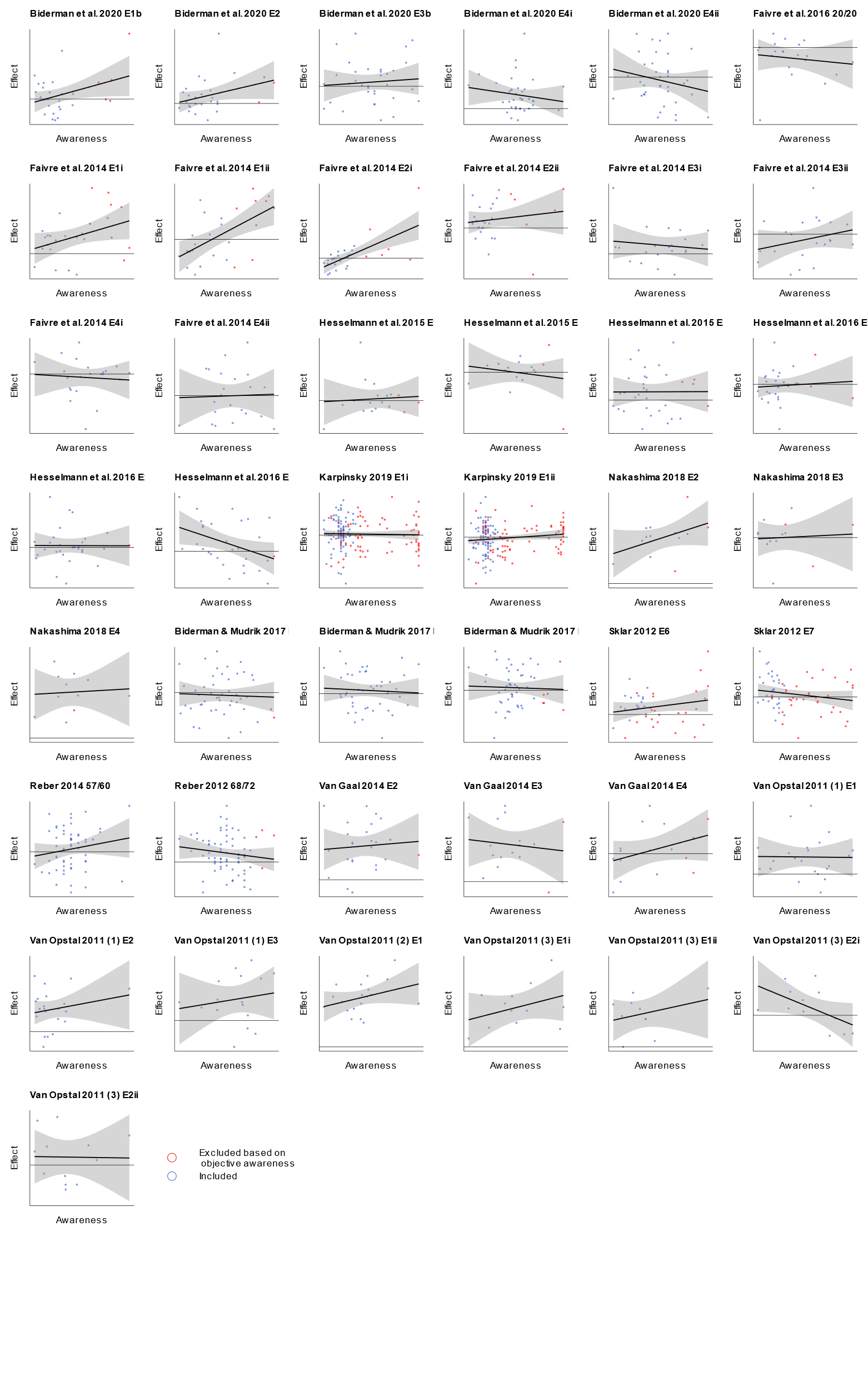
Supplementary Figure 1. Each scatter plot depicts the relation between awareness and effect scores for each dataset (N = 43). Blue, red, and gray dots indicate participants who were included, excluded based on high awareness score, and excluded based on other reasons, respectively. Black lines are linear regression lines, with gray areas denoting standard error.

**2. Existing solutions to the RttM problem in studying unconscious processing**

So far, different solutions have been proposed to the problem of regression to the mean (RttM) in unconscious processing studies. In the main paper, we only refer to them briefly, for brevity and ease of reading. Here, we provide a more detailed description of these solutions, to complement the results reported in the main text.

**Compatibility with RttM (Shanks, 2017)**

The basis for Shanks’ solution is predicting the RttM-expected scaled unconscious effect in the included group (the group of participants selected post-hoc according to their awareness scores), using the regression line of the entire group (with the function$Z\left( E \right)=r*Z\left( A \right)$, where $E$ is the mean unconscious effect and $A$ is the mean performance in the awareness measure, both calculated for the included group, $r$ is the Pearson correlation between $E$ and $A$ calculated for the entire sample, and Z is a scaling transformation)^^[[1]](#footnote-1)^^. Then, the critical question is whether the predicted effect of the included group according to linear regression, $Z(E)$, exceeds a 95% Confidence Interval (CIs) around the observed effect in the included group. The test asks if the predicted effect falls within the confidence interval of the observed effect. If so, it is deemed consistent with RttM, and accordingly considered not to be a genuine demonstration of unconscious processing.

We first applied this test to all the datasets we acquired. Quite strikingly, all of them failed it (Supplementary Figure. 2; see also Figure 6 in the Main text). This can imply that all reported effects are driven by - or at least compatible with - RttM. An alternative interpretation, however, is that the test itself might not be adequate or sensitive enough to detect effects even when they exist. To examine if this is the case, we conducted a series of simulations. Simulation studies are ideal for arbitrating between the two possible interpretations because we can define the ground truth ourselves (while for the real data, we only have the observed results, and cannot determine if a real effect exists or not).


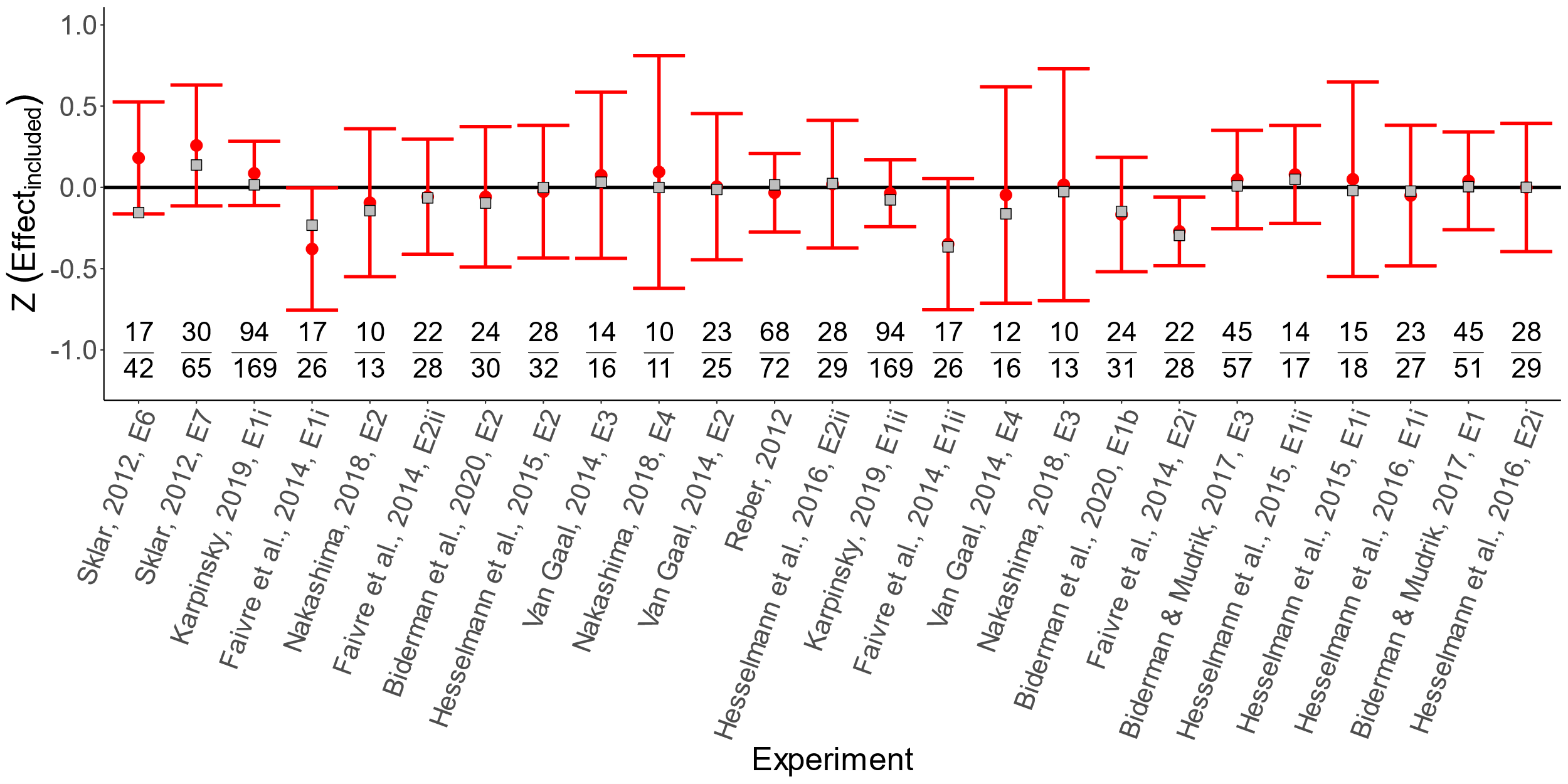


Supplementary Figure 2. Compatibility with RttM test results for 25 effects (eighteen additional effects were excluded from this analysis because no participants were excluded from the analysis based on awareness scores). On the left (white background) are experiments that reported positive results. On the right (gray background) are experiments that reported null results. Circles denote the observed effects, and error bars denote 95% CIs. Gray squares represent the RttM-predicted effects. When the circles and the error bars are colored in red, the effect is compatible with RttM, as was the case for all datasets. All values are normalized using Z-scoring, as suggested by Shanks, 2017. Numbers above the x-axis represent the fraction of participants who were included, with the numerator indicating the number of included participants and the denominator indicating the number of collected participants. The labels below the x-axis represent the name of the tested effect, starting with the name of the paper, followed by the experiment number, and - where needed - the index number of the specific effect tested, denoted by Roman numerals. Note that when $Z\left( E \right)$ equals zero, the predicted effect coincides perfectly with the observed effect, regardless of its actual magnitude (which may be below or above zero).

We performed a controlled simulation study to investigate the proposed test’s ability to identify unconscious processing, using the general testing framework described in the main text. We used a bootstrapping procedure with 10,000 iterations to estimate the confidence interval (CI) around the scaled observed effect score, and tested if the predicted effect $Z(E)$ exceeds this CI. The simulation clearly demonstrated the lack of sensitivity of the test to unconscious effects (Figure 8 in the Main text). That is, when a genuine true effect exists, less than 5% of the simulated datasets were deemed incompatible with RttM, and thus reflecting unconscious processing.

What, then, is the problem that prevents this test from finding these true effects? We argue that the main issue is that the proposed method does not test for an unconscious effect that is not driven by RttM, but rather a nonlinear relationship between awareness and effect, relying on linear regression assumptions, which might not be justified here (both the assumption of linearity, and the assumption of homoscedasticity which is violated in the common case of using 2AFC tasks to measure awareness). The key formula that underlies the method, $Z\left( E \right)=r*Z\left( A \right)$, is based on an assumed linear relationship between the effect and awareness measures. The formula holds true as long as the assumptions of linear regression are met, and importantly, regardless of whether there is unconscious processing. To demonstrate that this is the case, we adjusted our general testing framework to conform with the assumptions of linear regression (Supplementary Figure 3), sampling true and error scores from normal distributions for both awareness ($true \sim N\left( .5,.1 \right)$ and $error \sim N(0,.1)$) and effect scores (where true scores are defined as a function of the true awareness scores and $error \sim N(0,.1)$), setting sample size to N = 1000 to allow the test to hold enough power to detect non-linearity. Then we simulated four different scenarios, where the effect is entirely determined by awareness levels and with no unconscious effect, yet the relations are not linear (see Supplementary Table 1 for details about the specific transformations that were used): square root, quadratic, logistic and exponential.

| Relation | Formula ($A, E$) |
| --- | --- |
| Linear | $E=c* A$ |
| Square root | $E=c* \sqrt{A}$ |
| Quadratic | $E=c* A^{2}$ |
| Logistic | $E=c*\frac{L}{1+e^{-k (A-\frac{1- x_{0}}{2})}}$  Where $L=1, k=20, x_{0}= chance= .5$ |
| Exponential | $E=c*(2^{A}-1)$ |

**Supplementary Table 1. Simulations framework relations between awareness and effects.** For all relations, $A$ and E denote the true awareness and effect scores, respectively, and $c$ was defined to adjust effect scores according to the controlled simulation parameter that controls for the effect size of the participants (e.g., an effect size of Cohen's d = 1.2 for a fully aware participant in our simulations). When simulating the 'Independent Sources' relation (see section 'Compatibility with RttM'), awareness and effect scores were sampled independently.

Given the results in Supplementary Figure 3, it is clear that although no unconscious effect exists, the test yielded a ‘significant’ (or RttM-incompatible) result for different non-linear relations. Under this scenario, the results clearly show that the test differentiates between linear and non-linear relations, while completely lacking sensitivity to unconscious effects when the relations do not deviate sufficiently from linearity.


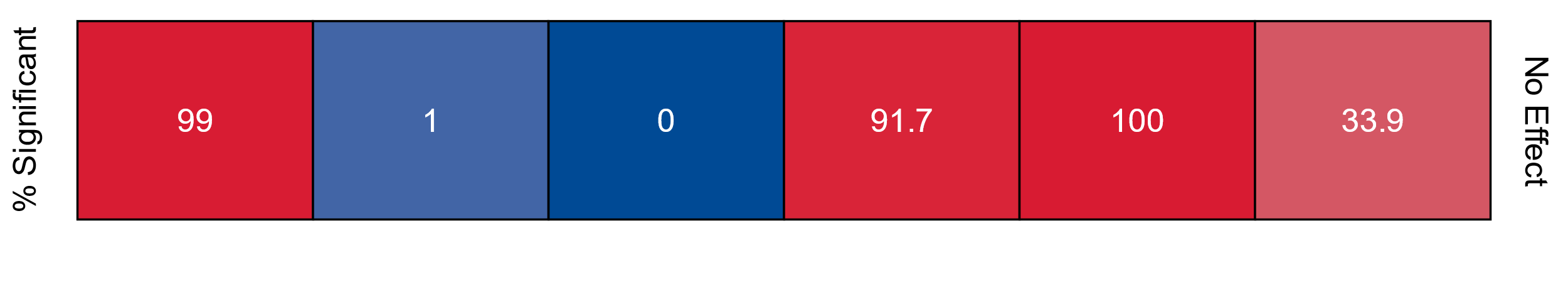

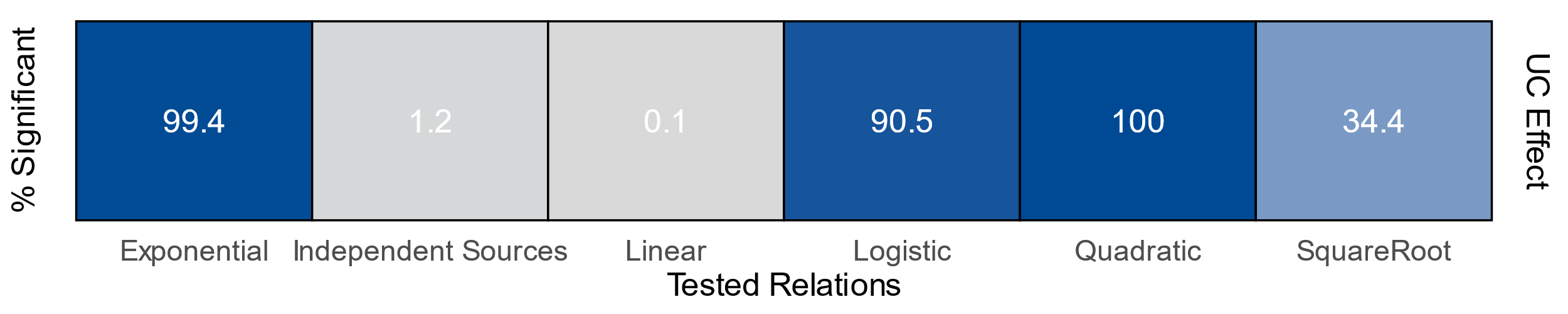


Supplementary Figure 3. Simulation results of the RttM compatibility solution (Shanks, 2017), generated according to a Gaussian noise model, and a fixed sample size of N = 1000. The label on each tile denotes the percent of significant effects across 1000 iterations. Upper panel: false-positives rate for different relations between awareness scores and the observed effects. Red and blue cells indicate that the proportion of false-positive effects is above, or below 5%, respectively (i.e, the number of simulated datasets the solution yielded an RttM-incompatible, when no unconscious effect exists). Lower Panel: power estimation for the same relations between awareness scores and the observed effects, when an unconscious effect exists (the effect scores of participants with true awareness score of zero were .5). Cell colors indicate the proportion of true-positive effects. The results show the sensitivity of the test to deviations from a linear model (in the 'Independent Sources' condition no relation exists between the measures), and completely lacks sensitivity to unconscious effects (similar results across panels, though one depicts a case where an effect exists and the other a case when it does not).

**Generative Bayesian framework (Goldstein et al., 2021)**

Another solution builds on the work by Matzke et al. (2017), and tackles the problem by modeling the hierarchical structure of the data. Unlike the 'Compatibility with RttM' solution suggested by Shanks (2017), Goldstein's solution directly addresses the issue of measurement error in awareness and effect measures by estimating the true regression line between these measures. Specifically, it involves estimating the true awareness and effect scores as well as the uncertainty in these measures for each of the participants. Then, these estimates are given as input to Matzke's model, where uncertainty estimates are used to correct for measurement error, computing a distribution of credible intercept estimates for a regression line of awareness on effect scores, to measure the effect of unaware participants (the effect of participants with zero awareness score, as awareness scores are re-scaled around chance level performance). The crux of the suggested solution is that the final estimate for the unconscious effect is extrapolated from the adjusted regression line describing the relation between awareness scores and the true effect, accounting for uncertainty in both these measures.

Crucially, this solution also depends on the unconscious effect being linearly related to the objective awareness scores and assumes homoscedasticity (the authors acknowledged that this assumption is likely to be violated, and used simulations to show robustness to its violation). Here, linearity is assumed explicitly, though the authors state that the solution could be modified to account for any specific non-linear relations, meaning that in any case, researchers should have some model of the relations between the effect and awareness, to use this solution. The authors explain that the linearity assumption makes sense, as the two factors depend on the percentage of trials in which a given participant is aware of the stimulus. However, given that, as we explain in the main text, the linearity assumption has already been criticized (Dosher, 1998; Klauer et al., 1998; Merikle & Reingold, 1998; Rouder et al., 2007), and for brevity, we decided not to test this solution and suffice with suggested solutions that do not explicitly make this assumption.

**Bayesian awareness categorization technique (BACT; Leganes-Fonteneau et al., 2021)**

The BACT (Bayesian awareness categorization technique) was suggested by Leganes-Fonteneau et al. (2021) to test claims for implicit, rather than unconscious or subliminal, effects. Yet the same rationale applies also for unconscious effects: using Bayes Factors (BFs; Jeffreys, 1939) to directly classify each participant as aware ($B_{aware}$), unaware ($B_{unaware})$, or insensitive ($B_{insensitive}),$ using the method ‘Bayesian Analysis Categorization Technique’, or BACT). Thus, as opposed to most other approaches, this solution does not rely on Null Hypothesis Significance Testing (NHST; e.g., binomial test), and does not focus on group-level testing of awareness measures against chance level performance, but on individual participants. Also, different from the two other solutions reviewed above, and akin to our solution, it does not rely on any assumption about the relation between awareness and effect, as it only focuses on the awareness scores. After classifying participants as $B_{aware}$, $B_{insensitive}$, or $B_{unaware}$ (the terms were taken from the original paper), the effect scores of participants deemed unaware are used to test for unconscious processing, while aware and insensitive’ participants are excluded. The authors validated this technique by using previously collected data to show that BACT yields reliable results, so that repetitive random splitting of awareness scores, results in stable $B_{unaware}$ categorizations for participants, with the average awareness score of the two halves of these participants being below chance level performance. This was taken as evidence for preventing RttM effects, as the latter is expected to lead to inconsistent results (i.e., participants categorized as unaware according to one random half, should be classified as aware according to the complementary half). Then, a t-test on effect scores for the participants classified as $B_{unaware}$ is taken as an uncontaminated estimation of the group-level unconscious effect. Thus, the authors concluded that this method prevents RttM effects on awareness measures, and suggested BACT as a solution to the contamination of unconscious samples by misclassifying aware subjects as unaware due to RttM.

Notably, the results of the BACT depend on *an adequate choice of two parameters*: first, a prior on the maximal awareness measure expected for a subjectively unaware participant. Second, a BF cutoff threshold for the classification of each participant’s BF as aware or unaware, or inconclusive. Specifically, the authors explain that the former parameter should be chosen based on prior studies, while the default choice suggested for the latter parameter is BF of 3-1/3, held to correspond to the common significance level in NHST tests (p < .05) (Dienes, 2014; Jeffreys, 1939). The authors offer a more conservative cutoff threshold selection procedure in which the most liberal BF threshold that satisfies the requirement to prevent RttM is chosen, in an iterative process going over different cutoffs from a stringent cutoff of BF = 6-1/6 until resolution.

We applied this solution to the datasets we acquired^^[[2]](#footnote-2)^^, setting the prior according to the minimal proportion of correct responses yielding a significant above-chance performance determined by a binomial test (one-tailed, alpha = 0.05; this mimics the objectively defined threshold for (un)awareness used in unconscious processing studies, to retain a type-1 error rate of 5%), and a BF cutoff threshold of 3-1/3 (note that only 19 datasets included trial by trial data, and collected a 2AFC measure of awareness necessary for applying the solution). As Figure 6 in the main text shows, a two-sided t-test of the effects of participants classified as $B_{unaware}$ yielded no significant effects. Importantly, applying the solution resulted in the exclusion of almost all participants (no participant was classified as unaware on 32% of the datasets, M = 1, SD =0.94). This can be explained in three ways: first, it might be that almost all participants in these studies were indeed aware. Second, the awareness measures in these studies might not have been sensitive enough to classify the awareness of individual participants. And third, the Leganes-Fonteneau test might simply be too strict^^[[3]](#footnote-3)^^*.*


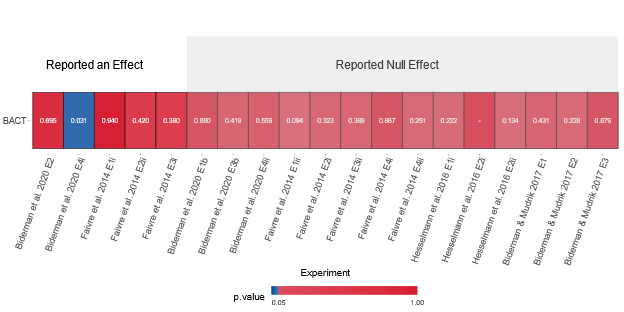


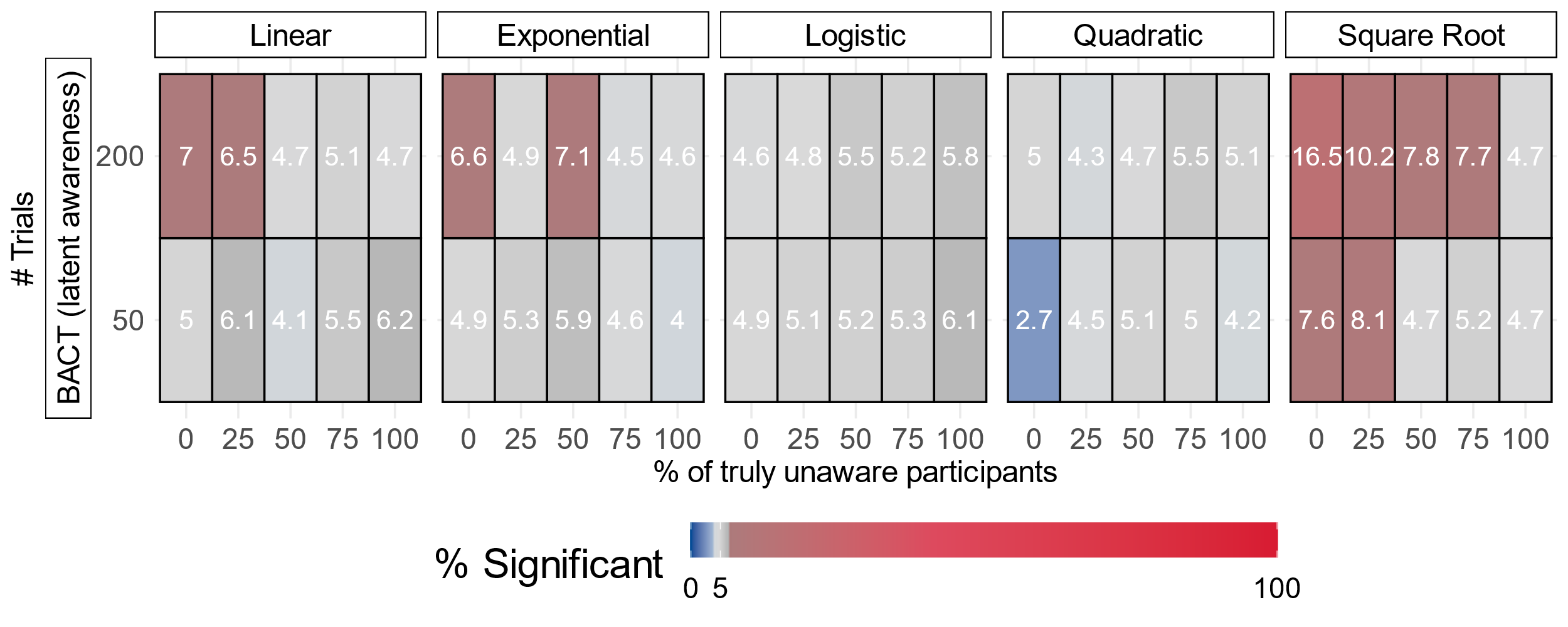


Supplementary Figure 4. Testing the BACT solution. Upper panel: Results of applying the solution to the acquired datasets (N = 19; the same experiments used in Figure 6 in the main text), with a prior set according to the original threshold used by the authors of each study. Cells report p-values for the observed effect of the participants deemed as unaware by the solution. Blue cells denote significant results (alpha = .05), red cells denote non-significant results (experiments when for which the solution excluded too many participants to assess the significance of effects were marked with '-'). Lower panel: Results of applying the solution on simulated data with the prior on the latent variable of average awareness score of truly aware participants set to 62% correct. Cells denote the false positive rates of the solution: the percent of significant effects found under different simulation parameter combinations where no unconscious effect exists. These parameters include the relations between awareness and effects (vertical panels), percent of truly unaware participants (x-axis) and the number of trials used to measure awareness (y-axis). Red cells indicate that the proportion of false-positive effects exceeds 5% (higher than the upper bound of a 95% confidence interval around 5% according to a random process with p = .05), gray cells denote cases in which this proportion was within the 95% CI around 5%, and blue cells denote cases in which this proportion was below 5%.

Akin to the above section, we again examined these alternative explanations using simulations, using the same prior and BF criterion used in the analysis of the empirical datasets. Then, we ran a one-sided t-test on the included sample (participants classified as $B_{unaware}$) to determine if an unconscious effect exists. The simulation suggested that the power of the solution is extremely low (with 200 trials and all participants being truly unaware, ranging between 17.6 and 19.1%, and even lower when there are also aware participants in the sample; Main text, Figure 8). On the positive side, the false positive rate (type 1 error) is within the expected values of 0.05 (with 200 trials and all participants being truly unaware ranging between 3.4 and 5.5%; yet note a single exception, in the 'Square Root' relation condition when all of the participants are aware and 50 trials are used; Main text, Figure 7). Thus, although the solution does well in screening out false results, its ability to detect true effects when those exist is very low due to the high exclusion rate for unaware participants^^[[4]](#footnote-4)^^ (see Supplementary Figure 5).


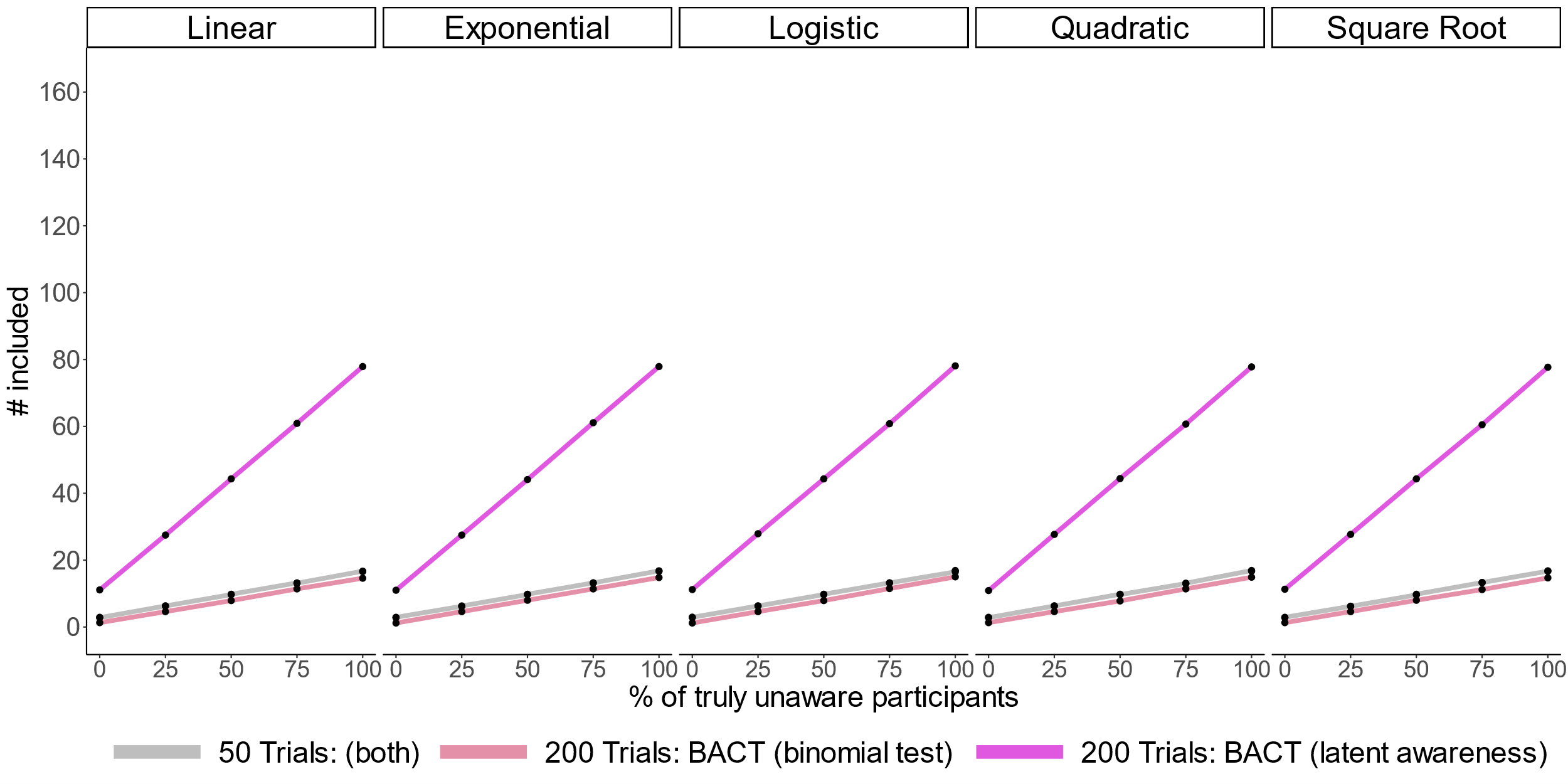


Supplementary Figure 5. The average number of included participants (classified as $B_{unaware}$) according to the BACT with two different prior settings when estimating the power of the solution in detecting unconscious effects in simulated datasets (see Figure 8 in the main text), with 200 trials (pink) and 50 trials (gray). In one case (dark shade), the prior was set according to the latent variable of the average awareness score of truly aware participants, defined as 62% correct. In the other case (light shade), the prior was according to a binomial test (see Figure 7 in the main text for the respective results with regard to testing for unconscious effects). For 50 trials, the results of the two cases completely overlapped, so there is no shade differentiation in the figure.

Another limitation of this solution is that it is solely based on the awareness measure, which raises again the problem of reliability discussed in the main text: if the measure is not reliable to begin with, how can we use it as means to exclude participants? The authors somewhat mitigate this problem by adopting a strict approach in searching for positive evidence for the null. However, as we have seen, this leads to the exclusion of the vast majority of participants.

**Split solution (Shanks, 2017)**

Another suggestion made by Shanks 2017, which can be formalized into a solution for the problem of RttM, is showing consistent classifications of group-level (un)awareness across two independent measures of awareness. Importantly, adding a second independent awareness measure might lead to underestimating awareness, due to increasing task and memory demands (Newell & Shanks, 2014). To overcome these issues, and to enable a retrospective analysis of previously acquired results, Shanks suggested random splitting of awareness data (e.g., to odd and even halves; Shanks, 2017), where one half of the data can be used as an additional, independent, awareness measure. The rationale here is to look for a sub-sample of participants for which, awareness scores are at chance for both halves of the trials, and then test if the observed effect is significant when using this sub-sample only. Here, we implemented this suggestion, to test its performance and compare it with other suggested solutions.

We implemented a Bayes Factor based, awareness testing procedure: First, we sorted N participants according to their awareness scores. Then, we split the awareness trials of participants into two random halves and used a Bayesian t-test iteratively (with a default prior of $r=\frac{\sqrt{2}}{2}\boldsymbol{)}$to test one half of the awareness scores of a subsample of the N-$i$ lowest awareness participants (where $i$denotes the iteration index). This gradual approach, starting from the full sample size and decreasing it by 1, where each time removing the participant with the highest awareness score, allowed us to find the largest sample that can be considered unaware: Once we found evidence for chance level performance in the first half ($BF_{01}$ > 3), we validated that for the same sub-sample (tested in that iteration), awareness scores of the other half of the trials also yield evidence for chance level performance (using the same criteria). Having found a subsample that passes both tests, we used a t-test to test for significant effects in this subgroup.

As the results show, the test is highly non-specific. This is demonstrated by showing a high false-positives rate in our simulations study, reaching 75.6% in the different simulation conditions (Figure 8, main text).

**Campbell & Kenny solution (Campbell & Kenny, 1999; Rothkirch, et al., 2022)**

Another correction method for RttM effects was suggested by Campbell & Kenny (Campbell & Kenny, 1999). Specifically, they suggested a formula that corrects the observed scores for a given measure by shrinking them according to both the group mean and the reliability of the measure: $C_{O}=M_{O}-r_{xx}\left( M_{0}-O \right).$Where $C_{O}$ is a vector of corrected observed scores, $M_{O}$ is the group-level mean of observed scores, $r_{xx}$ is the reliability of the measure used to obtain the observed scores, and $O$ is a vector of observed scores (see also Rothkirch et al., 2022 for a graphic illustration of this formula). We used a similar procedure to the one used for the 'Split solution' (described above), to compare the performance of this method with other solutions. We integrated the correction formula within the procedure, calculated the reliability of the awareness measure using Spearman-Brown corrected split-half reliability measure estimated across the entire sample (using 5000 random split halves), and then used a Bayesian t-test applied to the corrected scores of participants calculated based on all of the subsample's data (as opposed to using only one random half, as was described for the 'Split solution'). Again, once a subsample showed evidence for corrected awareness scores being at chance, a t-test was used to test for a significant effect in this subsample.

Similarly to the 'Split solution', the Campbell & Kenny solution was found to suffer from a lack of specificity (although to a lesser degree), which was evident by the inflated false-positive rate found in the simulations study (Figure 7).

**3. Reliability simulation - Exploring the relations between reliability, number of trials, and the proportion of unconscious subjects, for awareness measures**

In this simulation, the sample size was fixed at 40 participants, which is the average sample size in the collected datasets. In each iteration, we examined the Spearman-Brown corrected reliability (estimated across 5000 random splits) for 2AFC awareness measures including 30, 50, 200, and 500 trials, from samples of 0, 50, 75, 90, 95, and 100% truly unaware participants (the awareness scores of the complementary % of aware participants were sampled from a half-normal distribution with SD of .15) for 1000 iterations for each condition.

**4. Erroneously inferring unconscious processing from fully conscious samples**

The simulations whose results are reported in Figure 8 in the main text explore a range of cases where the percentile of unaware participants varied between 25% to 100%. Here, we complemented that simulation by also testing the different solutions on a case where the entire sample of participants was aware (i.e., 0% unaware participants). In this scenario where the is no unconscious effect (as all participants are aware), NPB was the only one for which the false-positive rate was lower than 5% in all conditions (see Supplementary Figure 6).


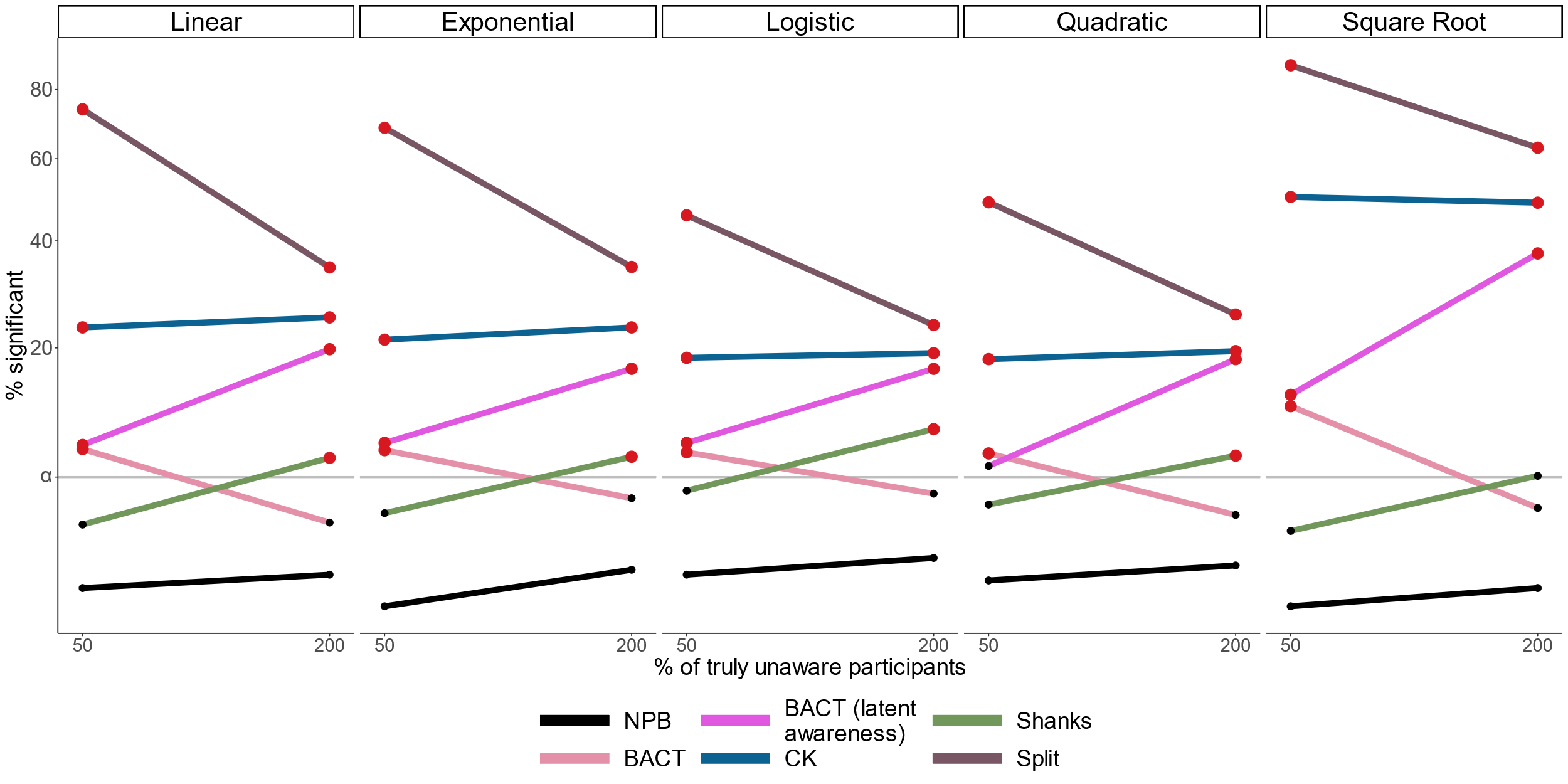


Supplementary Figure 6. Simulation results for a situation in which there is an unconscious effect, but the simulated sample only includes aware participants (the complementary condition of Figure 8, in the main text, including also the results of the BACT with a prior set according to the latent variable of average awareness of aware participants, depicted in magenta). As in Figure 7 in the main text, red points indicate that the proportion of significant effects exceeds 5% (reflecting false inference of unconscious processing based on fully aware samples). A square root scale was used for the y-axis to facilitate comparing the solutions around the $\alpha=0.05$ threshold requirement.

**5. Comparing the solutions using an area under the curve (AUC) analysis**

To complement the specificity and sensitivity analysis described in the main text (see Figure 7,8), we report a comparison that combines both aspects, by measuring the AUC for the different solutions, using the same simulation results. In this analysis, we quantify the area under a receiver operating curve (ROC) for the classification of simulation iterations as either containing an effect or not. To that end, we defined the hit rates as the rate of significant results of each solution under each tested condition when an effect existed in the data, and the false alarm rates as the rate of significant results of each solution under each tested condition when an effect was absent. Then, using the 'pROC' R package (Robin et al., 2011), we calculated the AUC for each solution under each simulated condition, classifying the existence of an effect according to the obtained p-values. The results clearly show higher AUC for the Split and CK solutions over the remaining solutions when some portion of the sample is truly aware (see Supplementary Figure 7). These results are driven by the high sensitivity of these solutions to effects (see Figure 8 in the main text). Yet in the context of unconscious processing studies, and the original criticism regarding the potential of contamination by conscious processing, this sensitivity might not be a blessing, as it is not specific: the goal of these solutions is to detect genuine unconscious effects, as opposed to false effects driven by conscious participants. Thus, if these solutions detect effects also in the latter case, they seem less preferable for the current purposes.

To further demonstrate this point, we examined the AUCs in conditions where all participants were either truly aware or unaware. In the former cases, where by design there is no unconscious effect in the data, both the Split and CK solutions show higher AUCs compared with NPB and BACT (Supplementary Figure 7; the leftmost points, colored in red). Hence, in these conditions, the higher AUCs of the solutions stem from sensitivity to conscious effects rather than to unconscious ones, echoing the inflated false-positive rate across conditions of these solutions (see Figure 7 in the Main text). In contrast, when all participants were truly unaware (Supplementary Figure 7; the rightmost points), NPB shows the highest AUCs compared with all other solutions.


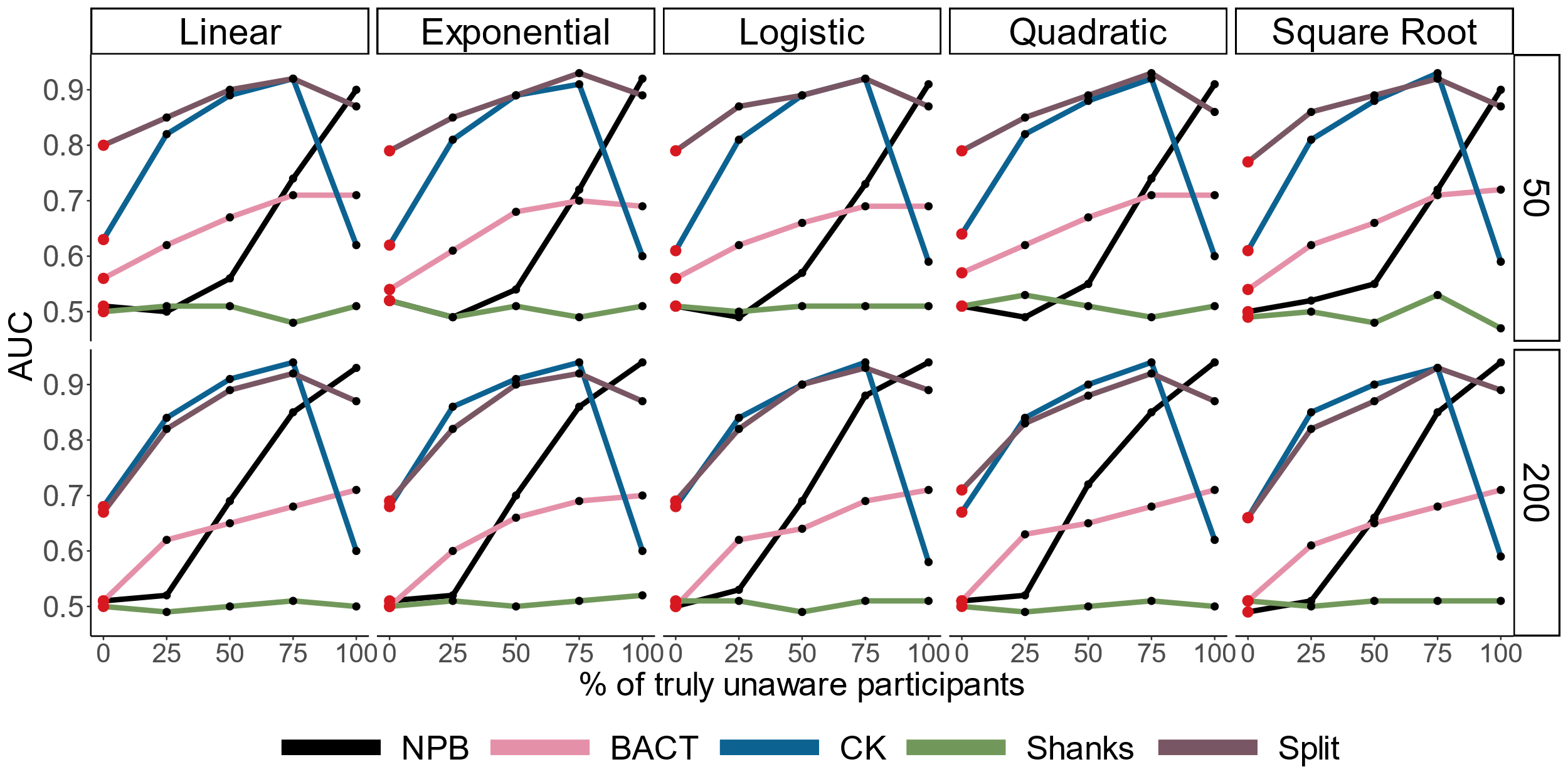


Supplementary Figure 7. The area under the curve (AUC) of the different solutions, for all simulated conditions. Lines denote the AUC of each solution in each condition. The leftmost points, colored in red, indicate the AUC of the solutions when all participants are truly aware. Thus, the higher AUC for both Split and CK across all relations compared with NPB indicates that unlike NPB, Split and CK are affected by aware participants (see also Supplementary Figure 6, and the Square Root condition of Figure 7 in the main text showing similar results across relation conditions). Similarly, when there is some proportion of aware participants, Split and CK show higher AUCs compared with NPB, except when 100% of the participants are truly unaware, where NPB show the highest AUCs,

**6. Supplementary Equations**

For all equations lower and upper case $e$ and $a$ denote participant and group level, effect and awareness scores, respectively.

**Supplementary Equation 1:**

$$e_{boot_{i,j}}=\left\{ \begin{aligned} e_{obs_{i}}, a_{obs_{i}}>h_{adj} \\ e_{perm_{i,j}}, a_{obs_{i}}\leq h_{adj} \end{aligned} \right.$$

Where $e_{boot_{i,j}}$denotes the bootstrapped effect of the i-th participant on the j-th surrogate dataset, $e_{obs_{i}}$ and $a_{obs_{i}}$ denotes the observed effect and awareness score of the i-th participant and $e_{perm_{i,j}}$ is the effect calculated for the i-th participant at the j-th random permutation of condition labels.

**Supplementary Equation 2:**

$${a_{boot}}_{i,j}\sim Binomial(a_{obs_{i}},N_{i})$$

Where $a_{boot_{i,j}}$ denotes the bootstrapped awareness score of the i-th participant on the j-th surrogate dataset, and $a_{obs_{i}}$ and $N_{i}$ denotes the awareness score and the number of trials used to measure awareness for the i-th participant.

.

**Supplementary Equation 3:**

$$I_{boot_{j}}=\{i | a_{boot_{i,j}}<h\}$$

Where $I_{boot_{j}}$ denotes the group of included participants on the j-th surrogate dataset based on the bootstrapped awareness scores, and $h$ denotes the *initial threshold* used by the authors as an exclusion threshold.

**Supplementary Equation 4:**

$$E_{boot_{j}}= \frac{1}{|I_{boot_{j}}|}\sum_{i \in{I_{boot}}_{j}} e_{boot_{i,j}}$$

Where $E_{boot_{j}}$ denotes the group-level effect for the j-th surrogate dataset.

**Supplementary Equation 5:**

$$I_{obs}=\{i | a_{obs_{i}}<h\}$$

Where $I_{obs}$ denotes the included participants based on the observed awareness scores and$h$ (the *initial threshold*).

**Supplementary Equation 6:**

$$E_{obs}= \frac{1}{|I_{obs}|}\sum_{i \in I_{obs}} e_{obs_{i}}$$

Where $E_{obs}$denotes the average group-level effect according to the observed effect scores of included participants (according to their observed awareness scores, $I_{obs}$).

**Supplementary Equation 7:**

$$p=P(E_{obs}> E_{boot})$$

Where $p$ denotes the p-value of the suggested test (the proportion of iterations where the observed group-level effect, $E_{obs}$, was greater than the RttM-mimicked effect, $E_{boot}$).

**References**

Robin, X., Turck, N., Hainard, A., Tiberti, N., Lisacek, F., Sanchez, J. C., & Müller, M. (2011). pROC: an open-source package for R and S+ to analyze and compare ROC curves. BMC bioinformatics, 12(1), 1-8.‏

1. Notably, Shanks refers to the unconscious effect as ‘Performance’ and the awareness measure score as ‘Awareness’, and uses different notations where X = 'Performance' and Y = 'Awareness', yet we feel that this might be a bit confusing, as the score of the awareness measure represents participants’ performance in that task. Hence, we adopt a different terminology here, referring to ‘unconscious effect’ and ‘awareness score’. [↑](#footnote-ref-1)
2. The original implementation of the Leganes-Fonteneau solution ([https:// https://osf.io/p3t9c/](https://osf.io/mqgw4/?view_only=94f9f196a95a4e71b168f65988b66ed2)) expects the number of trials in the objective awareness test to be equal across participants. However, this was not the case in most of the datasets, since trials were excluded due to subjective reports of high visibility, or inaccurate target responses. Thus, we first adjusted the original implementation of the solution (note that the original implementation was written in MATLAB, thus we first converted it to R), and applied it to the 19 effects for which 2 Alternatives Forced Choice (2AFC) awareness measures were used, and trial-by-trial data was available. [↑](#footnote-ref-2)
3. See the upper panel of Supplementary Figure 4 for a similar analysis, where we set the prior for the solution according to the exclusion threshold set by the original authors (in contrast to our main analysis where the prior was set according to the upper bound of a binomial distribution around chance). Importantly, these more liberal priors resulted in the classification of more participants as unaware (M = 5.32, SD = 3.28), and only for 5% of the datasets no participants were classified as unaware. Here, three effects were found significant according to the solution, yet see the false-positives rate of the solution using similar prior settings on the lower panel of Supplementary Figure 4. [↑](#footnote-ref-3)
4. Notably, when the simulations are run using the default prior set in the original implementation of the solution (Leganes-Fonteneau et al., 2021), the results are worse than shown above, with a much higher false positive rate (type-1 error, being above 50% under the most extreme conditions of our testing framework; See the lower panel of Supplementary Figure 4 and Supplementary Figure 5 for the false-positive rate and number of included participants of the solution when setting the prior according to the latent variable of average true awareness scores of aware participants, as was done in the original paper). Indeed, the authors specifically say that researchers must provide an informed prior based on previous experiments. Yet, since we found an inflated false-positive rate using a more liberal threshold (see again the lower panel of Supplementary Figure 4), we set the prior according to this theoretical distribution rather than relying on prior results. [↑](#footnote-ref-4)
